# The diverse diaspora of CAM: a pole-to-pole sketch

**DOI:** 10.1093/aob/mcad067

**Published:** 2023-06-12

**Authors:** Joseph A M Holtum

**Affiliations:** College of Science and Engineering, James Cook University, Townsville, QLD4811, Australia

**Keywords:** C_3_ + CAM, C_4_ + CAM, CAM biogeography, CAM distribution, CAM evolution, crassulacean acid metabolism, facultative CAM, *Lecanopteris sinuosa* (Polypodiaceae), strong CAM

## Abstract

**Background:**

Crassulacean acid metabolism (CAM) photosynthesis is a successful adaptation that has evolved often in angiosperms, gymnosperms, ferns and lycophytes. Present in ~5 % of vascular plants, the CAM diaspora includes all continents apart from Antarctica. Species with CAM inhabit most landscapes colonized by vascular plants, from the Arctic Circle to Tierra del Fuego, from below sea level to 4800 m a.s.l., from rainforests to deserts. They have colonized terrestrial, epiphytic, lithophytic, palustrine and aquatic systems, developing perennial, annual or geophyte strategies that can be structurally arborescent, shrub, forb, cladode, epiphyte, vine or leafless with photosynthetic roots. CAM can enhance survival by conserving water, trapping carbon, reducing carbon loss and/or via photoprotection.

**Scope:**

This review assesses the phylogenetic diversity and historical biogeography of selected lineages with CAM, i.e. ferns, gymnosperms and eumagnoliids, Orchidaceae, Bromeliaceae, Crassulaceae, Euphorbiaceae, Aizoaceae, Portulacineae (Montiaceae, Basellaceae, Halophytaceae, Didiereaceae, Talinaceae, Portulacaceae, Anacampserotaceae and Cactaceae) and aquatics.

**Conclusions:**

Most extant CAM lineages diversified after the Oligocene/Miocene, as the planet dried and CO_2_ concentrations dropped. Radiations exploited changing ecological landscapes, including Andean emergence, Panamanian Isthmus closure, Sundaland emergence and submergence, changing climates and desertification. Evidence remains sparse for or against theories that CAM biochemistry tends to evolve before pronounced changes in anatomy and that CAM tends to be a culminating xerophytic trait. In perennial taxa, any form of CAM can occur depending upon the lineage and the habitat, although facultative CAM appears uncommon in epiphytes. CAM annuals lack strong CAM. In CAM annuals, C_3_ + CAM predominates, and inducible or facultative CAM is common.

## INTRODUCTION

Crassulacean acid metabolism (CAM) photosynthesis has evolved often in extant vascular plants, possibly between 68 and 104 times. Present in ~5 % of vascular plants, CAM is dispersed across ~320 genera in 38 families (Winter *et al*., 2021*a*; Gilman *et al*., 2023). Most CAM plants are angiosperms, but CAM is present in Lycophyta (~39 species), Pteridiophyta (~15 species), Cycadophyta (*Dioon edulis* only) and Gnetophyta (*Welwitschia mirabilis* only). Among angiosperms, CAM is expressed in some magnoliids, monocots and in the core eudicots (super-rosids and super-astrids) but is unknown in the basal eudicots (Buxales, Proteales, Ranunculales and Trochodendrales) and basal core eudicots of Gunnerales and Dilleniales.

Amongst this phylogenetic diversity, CAM intermingles with many other adaptations such that, apart from the presence of photosynthetic cells with large vacuoles and a metabolic cycle that accumulates malic acid at night, there is no such thing as a ‘typical’ plant with CAM nor a uniquely CAM habit or habitat. There is biogeographical structure related to the diversity and history of each lineage but, as a group, plants with CAM can be terrestrial, epiphytic, palustrine or aquatic. They can be arborescent, shrubs, massive cladodes, leafless with photosynthetic roots (Winter *et al*., 1985), perennials, annuals or geophytes. No parasitic plants are known with CAM, despite many such species having fleshy leaves. Only two carnivorous species with CAM are reported, *Brocchinia reducta* and *B. hechtioides* (Bromeliaceae; Givnish *et al*., 1997). Among plant–ant mutualistic epiphytes, which tend to inhabit nutrient-poor habitats, CAM is in the Apocynaceae (e.g. *Dischidia major*, Treseder *et al*., 1995), Bromeliaceae (e.g. *Aechmea bracteata*, *Neoregalia myrmecophila* and *Quesnelia arvensis*, Crayn *et al*., 2015), Orchidaceae (e.g. *Caularthron bilamellatum* and *Myrmecophila tibicinis*, Silvera *et al*., 2010), Rubiaceae (*Hydnophytum* species, *Myrmecodium* species and *Squamellaria* species; Winter *et al*., 1983; Tsen and Holtum, 2012; Chomicki and Renner, 2016) and Polypodiaceae (*Leconopteris sinuosa*, this manuscript).

### CAM plants are distributed globally

CAM taxa are present on all continents barring, possibly, Antarctica. Most large and small stem- and leaf-succulents with CAM inhabit seasonally dry and semi-arid regions of the tropics and subtropics, but many CAM species are native to the moist tropics and to temperate biomes, both wet and seasonally dry.

Plants with CAM grow close to the lowest and highest extremes of elevation, temperature, light and moisture supply tolerated by vascular plants. Growing close to the lowest part of any continent, near Sedom at the south of the Dead Sea, two *Mesembryanthemum* species exhibit substantial nocturnal acid accumulations of ≤171 mmol H^+^ kg^−1^ fresh weight (Winter *et al*., 1976). At close to the highest elevations inhabited by vascular plants, where water freezes for substantial parts of the year, the CAM cacti *Austrocylindropuntia floccosa*, *Oroya peruviana* and *Cumulopuntia ignescens* are known from 4700 to 4800 m a.s.l. in Peru (Keeley and Keeley, 1989; Hoxey, 2016). In the Bolivian Andes, aquatic *Isoëtes boliviensis*, *I. glacialis* and *I. herzogii* grow at 4450–4750 m a.s.l., where they exhibit sizeable nocturnal acidifications of 96–131 mmol H^+^ kg^−1^ fresh weight (Keeley, 1998*a*).

CAM is absent from plants on the Antarctic mainland [although the yet-to-be-tested fleshy-leaved *Colobanthus quitensis* (Caryophyllaceae) inhabits the Antarctic Peninsula], but species with CAM do grow in subpolar regions. In the Northern Hemisphere, weakly CAM eudicots, such as *Hylotelephium telephium*, *Sedum acre*, *S. annuum* and *Petrosedum rupestre* (Bender *et al*., 1973; Kluge, 1977; Kenyon *et al*., 1985), grow at ~70°N around the Arctic rim in Siberia and Norway. In British Columbia, Canada, in rain-shadow areas near the Rockies, strong-CAM *Opuntia fragilis* grows at 56°N near Fort St John and *O. × columbiana* grows at 51°N near Kamloops (POWO, 2022). The succulent C_3_ + CAM crassuloid *Rhodiola rosea* (= *Sedum rosea*), a European alpine species (Osmond *et al*., 1975), grows in Iceland and Novaya Zemla and has been collected from 77°N in Svalbad. Among aquatic species with CAM, the lycopsids *Isoëtes echinospora* and *I. lacustris* have been collected from 70°N in northern Norway, and the eudicot *Crassula aquatica* is known from 64°N. *Sagittaria cuneata*, growing at 68°N in the Northwest Territories of Canada, is probably the northernmost documented monocot with CAM, terrestrial or aquatic.

In the Southern Hemisphere, in South America, the monocotyledonous strong-CAM atmospheric epiphytic bromeliad, *Tillandsia castellanii* (−12.6 ‰; Crayn *et al*., 2015), grows at 52°S, the terrestrial eudicots *Austrocactus* sp. and *Pterocactus australis* grow at 53°S (POWO, 2022), and the aquatic *Isoëtes savatieri*, with a reported nocturnal leaf acidification of 204 mmol H^+^ kg^−1^ fresh mass (Keeley, 1998*a*), grows at 55°S in low coastal regions of Tierra del Fuego (Hickey *et al*., 2003). *Crassula moschata*, not yet tested for CAM, grows as far south as 54°S on sub-Antarctic islands, including Kerguelen and Macquarie Island.

The global distribution of massive terrestrial CAM species with fresh masses >~20 kg fresh mass is uneven. Such species, with succulent stems or leaves, are present in the New World, South, East and Northwest Africa, the Arabian Peninsula, and islands such as Madagascar, Socotra and the Canaries, but are essentially absent from Europe, Northeast Africa, Asia Minor, Asia and Oceania. An argument for the absence of large water-storing CAM succulents in Australia is that wet seasons over much of Australia are too unpredictable to refill plants reliably on an annual basis (Ellenberg, 1981; Holtum *et al*., 2016; Buckland *et al.*, 2022). In contrast, the seasonally dry places in the New World, southern Africa and Madagascar, where large aloës, agaves, bromeliads, cacti, euphorbs and didierids grow, experience reasonably predictable annual seasonal rainfalls. Lack of predictable rainfall cannot be the only explanation for the disjunct distributions of large succulents, because much of large succulent-depauperate Asia experiences relatively predictable rainfall, and introduced *Opuntia* and *Euphorbia* have established in some Australian landscapes (Mann, 1970; Osmond *et al*., 1979). Perhaps taxa with an evolutionary capacity to develop large succulent stems or leaves did not evolve or did not successfully disperse and establish in much of the globe. For example, the Agavioideae, Bromeliaceae and Cactaceae are restricted to the New World, *Aloë* and Didiereaceae are restricted to Africa, the Arabian Peninsula and Madagascar, and Asian *Euphorbia* are the result of a single dispersal event to India and Southeast Asia (Horn *et al*., 2014).

### The expression of CAM is variable

Carbon isotope ratios (δ^13^C values) of terrestrial and epiphytic plants with CAM that range from ~−8 ‰ to more negative than −30 ‰ are evidence of a ‘continuum’ of CAM phenotypes, among which dark CO_2_ fixation by phosphoenolpyrovate carboxylase can contribute anywhere between 100 and close to 0 % of whole-plant carbon gain (Winter and Holtum, 2002). This span translates to extremes of nocturnal acidification from ~300–400 mmol H^+^ kg^−1^ fresh mass in some arborescent *Clusia* (Borland *et al*., 1992) to levels close to the limits of detection of ~1–3 mmol H^+^ kg^−1^ fresh mass in some orchids and forbs (Silvera *et al*., 2005; Winter *et al*., 2019*a*, 2021*b*). This massive variation in the contribution of CAM to plant carbon composition indicates that the role of CAM differs among taxa.

There is often structure within the superficially continuous CAM isotopic spread, with a bimodal distribution of δ^13^C values around a minimum of ~−20 ‰ (Vogel, 1980; O’Leary, 1988; Crayn *et al*., 2015), a value consistent with 50 % of carbon being trapped at night and in the day (Winter and Holtum, 2002). The bimodal distribution implies that most species with CAM obtain either a majority or a minority of their carbon using the CAM pathway. One might thus predict that there are distinct ecological advantages of the strong-CAM and C_3_ + CAM conditions (Winter & Holtum, 2014) and little advantage to a state of obtaining 50 % of carbon gain during the light and during the dark. However, an intermediate δ^13^C value does not necessarily indicate that a plant obtains 50 % of its carbon from the C_3_ and CAM pathways on any single day. Such values might indicate a state whereby a plant initially obtains carbon via C_3_ photosynthesis and subsequently, when stressed or ageing, obtains carbon mainly via CAM (Niewiadomska and Borland, 2008; Winter and Holtum, 2014; Winter, 2019). Shifts from an initial more-negative C_3_ δ^13^C value towards a less-negative CAM-type signal reflect the relative contributions of the two modes of photosynthesis to the carbon mass in the organ tested. In some species, a C_3_-to-CAM switch is facultative, in that it can be reversed when stress is removed (Winter, 2019).

When the contribution of CAM to net carbon gain is <100 %, the remaining carbon is commonly provided by C_3_ photosynthesis. In *Portulaca* (Portulacaceae) and in at least one *Trianthema* (Aizoaceae), the contribution of carbon by the C_3_ pathway is replaced by C_4_ photosynthesis. In all probability, C_3_, C_4_ and CAM photosynthesis co-occur in the same plant in *Portulaca* (Lara *et al*., 2004; Holtum *et al*., 2017*b*; Ferrari *et al*., 2020; Gilman *et al*., 2022) and *Trianthema* (Winter *et al*., 2021*b*), although it has yet to be demonstrated that all three modes of photosynthesis are present in the same organs.

### CAM, temperature and elevation

In surveys of geographically diverse lineages, such as bromeliads, cacti, *Clusia*, Portulacineae and orchids, taxa with pronounced CAM are less common at higher elevations and in temperate regions, where growing seasons tend to be short (Arroyo *et al*., 1990; Holtum *et al*., 2004; Crayn *et al*., 2015; Torres-Morales *et al*., 2020; Pachon *et al*., 2022). The ecophysiology of these distribution anomalies between CAM, temperature, elevation and lineage has yet to be unpacked experimentally. It is also possible that low levels of isotopically invisible CAM might persist at higher elevations and in colder environments.

Regarding temperature, apart from the presence of the cold-sensitive enzyme pyruvate orthophosphate dikinase, which is expressed differentially across CAM lineages (Sugiyama *et al*., 1979; Holtum and Osmond, 1981), there is little evidence that the CAM cycle itself is overly sensitive to low temperatures. Perhaps, in cell-packed fleshy tissues, internal diffusion considerations become fundamentally limiting when temperatures drop, although if temperatures are low one might not expect rates of dissolved and gaseous carbon flow to be high. Nevertheless, high-elevation *Opuntia* fix CO_2_ at night when sub-epidermal temperatures are −3 °C (Keeley and Keeley, 1989). One could envisage roles for water-use-efficient CAM photosynthesis in environments where low temperatures reduce soil water potential and cold winds increase evapotranspiration.

If an elevational CAM species cut-off is not temperature related, why would CAM be restricted at high elevation? At higher elevations, dark carboxylation by phosphoenolpyrovate carboxylase is efficient at obtaining carbon when CO_2_ partial pressures are low, but lower ratios of intercellular-to-ambient CO_2_ mole fractions during daytime C_3_ photosynthesis result in increased carboxylation efficiency of rubisco at decreasing oxygen partial pressure (Farquhar and Wong, 1984; Cernusak *et al*., 2013). The C_3_ + CAM *Sempervivum montanum* survives extreme daytime temperature fluctuations in its European Central Alp exposed rocky habitats by closing stomata during the day and can obtain CO_2_ at night temperatures down to ~−2 °C, when ice formation begins (Wagner and Larcher, 1981). In the Northern Andes of Chile, CAM species tend to occupy lower elevational levels than their C_3_ counterparts and are more prevalent on the relatively drier western slopes (Arroyo *et al*., 1990).

### The origins of CAM

When CAM first evolved is not known. Because it is present in early lineages, such as *Isoëtes*, ferns and *Welwitschia*, it has been speculated that CAM might have appeared during the Cretaceous or Palaeocene, perhaps even as early as the Jurassic/Triassic in *Isoëtes* (Raven and Spicer, 1996; Keeley, 1998*a*). Recent phylogenetic analyses suggest later multiple independent origins. Several lineages have origins dated to the Oligocene, with major diversifications attributed to the mid–late Miocene and the Pliocene (Klak *et al*., 2004; Good-Avila *et al*., 2006; Bruyns *et al*., 2011; Arakaki *et al*., 2011; Givnish *et al*., 2014; Hancock *et al*., 2018; Wood *et al*., 2020). The diversification of lineages with CAM during epochs when the atmospheric [CO_2_] fell from ~1500 to <500 ppm (Rae *et al*., 2021) and when aridification increased supports the broad ecophysiological view that CAM photosynthesis is a CO_2_ pump co-opted convergently as a response to stresses associated with daytime CO_2_ limitation (Osmond, 1978; Keeley, 1998*a*). In terrestrial plants, CAM is associated with daytime stomatal closure, which increases water-use efficiency, and with increasing internal [CO_2_], which reduces photorespiration. In aquatic species, the increase in internal [CO_2_] during the day overcomes problems of low carbon content in waters and high resistances to CO_2_ diffusion, particularly across the unstirred leaf boundary layers (Keeley, 1998*a*), conditions that undoubtedly pre-dated Oligocene aridification.

The structure in CAM δ^13^C values across lineages provides a background for theories of the evolution of CAM and its phenotypes. Edwards and co-workers (Edwards and Ogburn, 2012; Edwards, 2019) considered a stepped trajectory from C_3_ through C_3_ + CAM to strong CAM, in which the expression of C_3_ + CAM requires CAM-type biochemistry and regulation but only a minimum of anatomical modification. They also proposed that the acquisition of strong CAM requires CAM biochemistry plus prominent anatomical structures that together enable the accumulation of high concentrations of malic acid in the vacuole and limit internal CO_2_ diffusion. If assembly of the basic biochemistry required for C_3_ + CAM photosynthesis, or perhaps even of facultative CAM (Yang *et al*., 2019), is more accessible in an evolutionary sense than assembling the distinct anatomy required for strong CAM, C_3_ + CAM stages might be expected to evolve before strong-CAM stages. The adoption of appropriate succulent structures and the emergence of the strong-CAM phenotype from subsets of C_3_ + CAM populations (or perhaps facultative-CAM populations) would thus be rate determining for any potential overall C_3_ to strong-CAM trajectory.

The model of more common or more evolutionarily accessible initial C_3_ + CAM states followed by strong-CAM states that are more difficult to achieve (Edwards, 2019) is consistent with the broad range of δ^13^C values of CAM plants and the typically bimodal distribution of the values. It predicts phylogenetically dispersed CAM-containing lineages, some of which express C_3_ + CAM only and some, probably fewer, which contain both C_3_ + CAM and strong-CAM taxa or are predominately strong CAM.

Interactions between C_3_ + CAM stages and their environment are likely to influence the subsequent selection for more pronounced CAM. A C_3_ + CAM stage might appear evolutionarily stable because the selection of strong-CAM phenotypes is not favoured from it (e.g. the Australian *Calandrinia*; Hancock *et al*., 2018, 2019). Alternatively, if genetic and environmental conditions favour selection, marked succulence and strong-CAM lines might emerge from C_3_ + CAM forbears that might or might not subsequently survive [e.g. in *Hechtia* (Bromeliaceae); Crayn *et al*., 2004].

A second model assumes that C_3_ and C_3_ + CAM phenotypes essentially do not differ (Bräutigam *et al*., 2017; Schiller and Bräutigam, 2021). Rather, all C_3_ plants have a capacity for some CAM-type acid accumulation, and the evolution of strong CAM from this basal C_3_ + CAM state simply requires a continuous and smooth upregulation of metabolism to a strong-CAM phenotype, presumably giving rise to a continuum of phenotypes along the way. Winter and Smith (2022) argued forcefully that the capacity for CAM-type acid accumulation at night is not a biochemical capability of C_3_ plants and that the switch to night-time malic acid accumulation and associated metabolic reprogramming that define CAM is a discrete evolutionary innovation.

## BIOGEOGRAPHIES OF SELECTED CAM LINEAGES

The lineages covered in this review of the distribution and diversity of CAM were, in the main, informed by the availability of suitable phylogenies that contained sufficient information on the distribution of CAM species within them. Two exceptions are the New World lineages Agavoideae and Clusiaceae, which are reviewed separately in the same issue of this journal (Heyduk *et al*., 2023; Luján *et al*., 2023).

### CAM in ferns

**Fig. 1.**
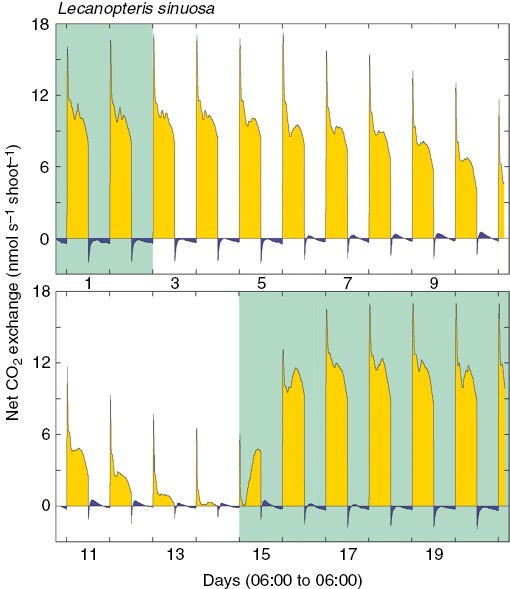
Demonstration of facultative CAM in the ant-fern *Lecanopteris sinuosa* (Polypodiaceae) during 20 days of net CO_2_ exchange by a frond attached to a piece of rhizome growing in commercial potting mix in a 0.65 L terracotta pot, with 12 h light (26 °C, 300 mmol m^−2^ s^−1^)–12 h dark (20 °C) periods. The flow rate was 1.5 L min^−1^. During the experiment, the plant was subjected to a watering–drying–rewatering cycle. The green background indicates days upon which the plant was watered to field capacity, and the white background indicates days when no water was supplied to the plant. Net CO_2_ exchange during the light is shown as yellow, whereas net CO_2_ exchange during the dark is shown as blue. Frond fresh mass was 0.611 g, and dry mass (4 days at 70 °C) was 0.104 g.

CAM is known in only 15 of ~13 000 species of ferns. CAM ferns include epiphytes, lithophytes and terrestrials within the most derived fern order, Polypodiales (PPG, 2016). Three CAM species are in the early-appearing polypod family Pteridaceae (*Polytaenium citrifolium*, *Haploteris flexuosa* and *Vittaria lineata*) (Carter and Martin, 1994; Martin *et al*., 2005; Schuettpelz *et al*., 2016) and 12 are in the later-appearing family, the Polypodiaceae (*Dictymia brownii*, *Lecanopteris sinuosa*, *Microsorum punctatum*, *Campyloneurum crassifolium*, two species of *Platycerium* and six species of *Pyrrosia*) (Hew and Wong, 1974; Wong and Hew, 1976; Sinclair, 1984; Keto *et al*., 1995; Holtum and Winter, 1999; Martin *et al*., 2005; Rut *et al*., 2008). Most ferns with CAM are C_3_ + CAM, but *Pyrrosia longifolia* exhibits strong CAM. Facultative CAM is reported here in *Lecanopteris sinuosa* (Fig. 1), an epiphytic tropical fern that hosts ants in its rhizomes. Although this is the first demonstration of facultative CAM in a fern, others can probably vary CAM expression, e.g. δ^13^C values for *Pyrrosia confluens* (= *P. dielsii*) vary between −17 and −25 ‰ (Winter *et al*., 1983; Messerschmid *et al*., 2021). It is likely that more species with CAM will be detected in *Lecanopteris*, *Microsorum*, *Platycerium* and *Pyrrosia*, and possibly also *Microgramma*.

The Platycerioideae, *Platycerium* and *Pyrrosia*, split from African and Madagascan polypod ancestors in the late Eocene, ~38 Mya, with *Pyrrosia* diverging in the late Oligocene, ~26 Mya (Wei *et al*., 2017). *Platycerium*, the elkhorn and staghorn ferns, is now a pantropical epiphytic/lithophytic genus of six species in Africa–Madagascar, ten species in Indochina–Malesia-New Guinea–Australia, and *P. andinum* in South America, which is nested within the African–Madagascan clade (Schneider, 2006). The splitting of Afro-American and Australasian *Platycerium* lineages post-dates the break-up of Gondwana, and thus long-distance dispersal with subsequent speciation is the inferred explanation for the current distribution of *Platycerium*. Although both the Afro-American and Australasian clades typically inhabit CAM-conducive seasonally dry tropical climates and the forests within them, CAM is reported only in two species from the Australasian clade, *P. veitchii* and *P. bifurcatum* (Holtum and Winter, 1999; Rut *et al*., 2008). Both exhibit characteristics of CAM cycling, in which net dark CO_2_ uptake is absent but nocturnal acidification occurs.

In the colonial nest-forming *P. veitchii*, a lithophyte of the Australian wet–dry tropics, CAM cycling is one of a suite of xerophytic traits that reduce water loss, extending the life of fronds. Colonially produced nests trap water and nutrients, lowering frond temperature and assisting survival on rocks that can attain 65 °C during the day (Keto *et al*., 1995).

*Pyrrosia*, the epiphytic and terrestrial fern genus with the most CAM species and the most pronounced expression of CAM, has a wide distribution in tropical and southern Africa, Madagascar, mainland Asia as far north as Siberia, Southeast Asia, New Guinea, Australia, New Zealand and various Pacific islands (Wei *et al*., 2017). Known CAM *Pyrrosia* are restricted to a single, not overly diverse *Pyrrosia* clade of South Asian, Indo-China and South China ancestry, with a divergence time in the mid-Miocene, ~14 Mya (Wei *et al*., 2017).

The emergence of CAM might be relatively recent in the polypod ferns, but some adaptations to water stress might have older origins, because early-diverging lineages, such as the microsoroids, which include *Microsorum* and *Lecanopteris*, inhabited xeric niches on rocks or isolated trees before the appearance of angiosperm-dominated tropical forests (Sundue *et al*., 2015; Testo and Sundue, 2016).

### CAM in gymnosperms and basal eudicots

The most basal seed plants with CAM are the gymnosperms, *Welwitschia mirabilis* (Gnetales) and *Dioon edule* (Cycadales), and Magnoliid angiosperms in *Peperomia* (Piperales) (Winter and Schramm, 1986; Holthe *et al*., 1992; Vovides *et al*., 2002; von Willert *et al*., 2005). For the gymnosperms, the amounts of acid accumulated are small, and net nocturnal CO_2_ uptake is negligible, if present.

It is uncertain whether *Welwitschia*, which diverged ~112 Mya (Ickert-Bond and Renner, 2016), is the oldest extant species with CAM, because CAM is unknown in its ancestors and it is unknown when CAM appeared in *Welwitschia*. Restricted to disjunct arid refuges in the Namib Desert, where rainfall is ~20–200 mm year^−1^, *Welwitschia* inhabits alluvial soils in or adjacent to small dry riverbeds, and terraces bordering larger dry riverbeds. *Welwitschia* probably comprises a Namibian and an Angolan subspecies (Leuenberger, 2001; Jürgens *et al*., 2021), which might have evolved following desertification that led to the extinction of intermediate populations during the Tertiary and Quaternary (Ickert-Bond and Renner, 2016; Jürgens *et al*., 2021). It is unknown whether CAM is present in both subspecies.

*Dioon edule*, one of 16 Mexican-endemic species of *Dioon* (Zamiaceae) (Gutiérrez-Ortega *et al*., 2018*a*), is the only cycad reported with CAM (Vovides *et al*., 2002). The near-threatened species, a member of a *Dioon* clade that shifted habitat towards arid zones during the Miocene (Gutiérrez-Ortega *et al*., 2018*b*), inhabits seasonally hot and dry tropical deciduous forests of Northeast Mexico (Vovides, 1990). The small, mainly dry-season contribution of CAM to net carbon gain is reflected in a C_3_-type δ^13^C value of −26.3 ± 0.4 ‰. Investigation for CAM activity of other *Dioon* species, such as *D. purpusii*, which has a δ^13^C value of −24.6 ± 0.8 ‰, and perhaps other cycad genera, is probably warranted.

Within the basal angiosperms, C_3_ + CAM, CAM cycling and facultative CAM are present in *Peperomia* (Piperaceae; Holthe *et al*., 1992), a species-rich genus with ~1411 mainly tropical and subtropical taxa (POWO, 2022). Of 93 species surveyed, 52 % exhibited CAM (Holthe *et al*., 1992; Ting *et al*., 1996). Most diverse in the Neotropics (>1200 species), particularly in the Andes and Amazonia, *Peperomia* also grow in Southeast Asia (~100 species), Africa (~20 species), Madagascar (~40 species) and Oceania (<20 species). Although the stem age of *Peperomia* is late Cretaceous, ~72 Mya (95 % highest posterior density: 66–79 Mya), and the crown age is early Eocene, ~54 Mya (95 % highest posterior density: 44–65 Mya), many lineages are younger, particularly those in Oceania (Lim *et al*., 2019).

*Peperomia* are unusual CAM plants, in that many inhabit shaded or dappled areas in wet tropical forests. Nevertheless, water-use-efficient CAM photosynthesis might be expected to be beneficial for survival of small plants that are mostly epiphytes, grow on rocks or in humus accumulated on rocks, grow in shallow soils or, uncommonly, are tuberous geophytes that grow in periodically locally dry sites. The relative contributions of CAM and hydrenchyma (cf. Males, 2017) to water-use efficiency and the successful diversification of *Peperomia* have yet to be quantified.

**Fig. 2.**
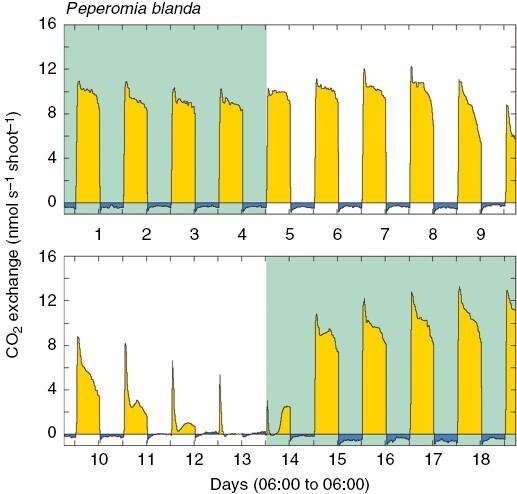
Demonstration of facultative CAM in *Peperomia blanda* (Piperaceae) during 18 days of net CO_2_ exchange by a shoot growing in commercial potting mix in a 0.8 L plastic pot, with 12 h light (26 °C, 300 mmol m^−2^ s^−1^)–12 h dark (20 °C) periods. The flow rate was 1.5 L min^−1^. During the experiment, the plant was subjected to a watering–drying–rewatering cycle. The green background indicates days upon which the plant was watered to field capacity, and the white background indicates days when no water was supplied to the plant. Net CO_2_ exchange during the light is shown as yellow, whereas net CO_2_ exchange during the dark is shown as blue. Shoot fresh mass was 4.564 g, and dry mass (4 days at 70 °C) was 0.223 g.

The biogeographical origins of *Peperomia* are unclear. Based on species diversity, a Neotropical origin is likely, but multiple introductions into Africa, Asia and Oceania are required if either a Neotropical ancestor or a Neotropical and African ancestor is postulated (Smith *et al*., 2008). In Oceania, *Peperomia* are the product of at least four colonization events out of the Neotropics (Lim *et al.*, 2019). A Hawaiian clade and a Pacific clade are sister groups to Central and South American species; a third *P. blanda* clade possibly has Caribbean origins; and a *P. tetraphylla* clade is sister to Neotropical taxa. Both *P. blanda* and *P. tetraphylla* exhibit CAM (Fig. 2). Unravelling whether colonization was westwards from tropical America, eastwards via Africa, or both, requires more intensive sampling. The complex frequent long-distance movement of *Peperomia* might be attributable to small and sticky seeds that are easily ingested by birds and readily stick to their feet and feathers. Dispersal of *Peperomia* seeds >5000 km by birds from South America to the Pacific Juan Fernandez Islands, then to Tristan de Cunha Island in the South Atlantic has been demonstrated (Valdebenito *et al*. 1990).

### CAM in the Orchidaceae

Orchids might have arisen in Australia ~112 Mya, spreading to the Neotropics via Antarctica by ~90 Mya, when extant orchid lineages began diverging (Givnish *et al*., 2015, 2016). Subsequent continental movements, the appearance and disappearance of land masses, fluctuations in sea level and climate, repeated transoceanic and short-distance dispersal, and expanding and contracting forest biomes resulted in their current global distribution (Givnish *et al*., 2015, 2016) and diversity, currently 729 genera containing ~28 000 species (POWO, 2022).

**Fig. 3.**
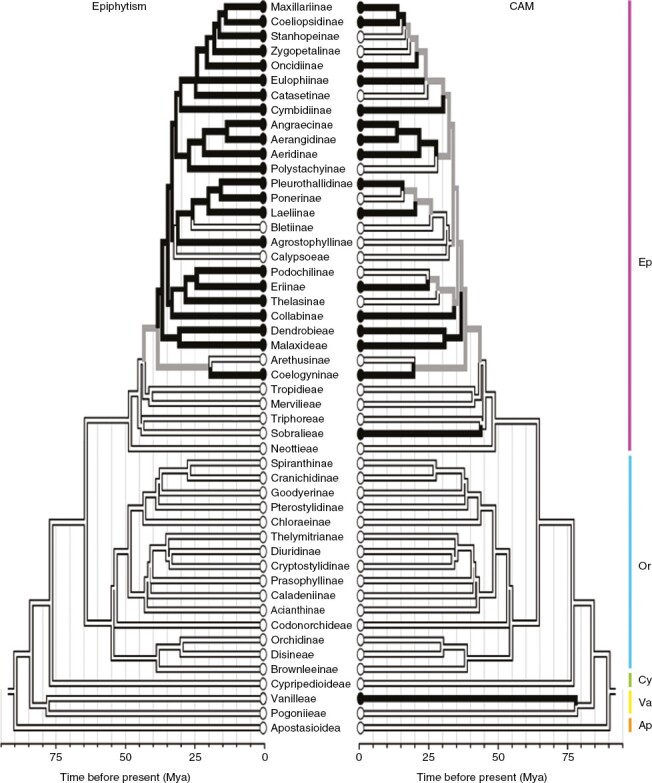
A chronogram of the Orchidaceae showing the occurrence of epiphytism and CAM. Branch colours reflect the ancestral presence (black) or absence (white) of CAM or epiphytism inferred using maximum parsimony; grey branches document uncertainty under maximum parsimony. The five subfamilies indicated by coloured vertical lines are Epidendroideae (Ep), Orchidoideae (Or), Cypripedioideae (Cy), Vanilloideae (Va) and Apostasioideae (Ap). The figure is an amalgum of fig. S2B and C in the paper by Givnish *et al*. (2014).

Most orchids are epiphytes (69 %; Zotz *et al*., 2021), a trait which, having evolved once no later than 35 Mya, has accelerated net diversification rates and has been lost several times (Fig. 3; Givnish *et al*., 2016). Initially a tropical lineage, some clades expanded out of the tropics (Givnish *et al*., 2016), and orchids now grow as far north as 72°N (*Listera cordata* from Greenland; *Pseudorchis albida*, northern Europe and Russia; GIBF, 2022) and as far south as 54°S (*Corybas* and *Nematoceras* spp., Macquarie Island; GIBF, 2022). Essentially all temperate orchids are considered terrestrial, although this postulate appears untested.

Currently, 95 orchid genera (~13 % of genera) are known to contain taxa with CAM (Gilman *et al*., 2023). CAM appears to have arisen at least four times at tribe and/or subtribe levels, three times in the Epidendroideae and once in Vanilleae (Fig. 3; Givnish *et al.*, 2015). When examined at finer phylogenetic scales, more CAM appearances are evident, e.g. CAM has evolved independently at least ten times among Neotropical genera (Silvera *et al*., 2009) and four times within the Eulophiinae (Bone *et al*., 2015*a*, *b*). In comparison to C_3_ photosynthesis, possession of CAM by orchid groups as a whole is associated with accelerated speciation, extinction and net diversification, perhaps a result of associations of CAM and epiphytism that enable adaptive radiations into new canopy spaces in both wetter and drier forests. In contrast, CAM had no significant effect on net diversification in bromeliads (Givnish *et al*., 2011), in which it occurs in epiphytes and in terrestrial lineages.

CAM orchids are epiphytic or lithophytic, with some exceptions (Fig. 3). The absolute number of orchid species with CAM is uncertain because the proportion of orchids surveyed in different regions varies. In studies of 1022 species from Panama and Costa Rica (Silvera *et al*., 2010) and 1079 species from Colombia (Torres-Morales *et al*., 2020), ~6 % of the global total, ~10 % exhibited strong CAM, including only a few terrestrial species, one of which was *Oecoclades maculata*, an introduction from West Africa. In smaller surveys, CAM was present in 58 % of 97 *Bulbophyllum* species from Asia (Hu *et al.*, 2022), 62 % of epiphytic orchids from Australian rainforests (Winter *et al*., 1983), 26 % of orchids in a mid- to high-elevation New Guinean rainforest transect (Earnshaw *et al*., 1987), 42 % of orchid species in moist lowland forest sites in Panama (Zotz and Ziegler, 1997) and 100 % in a Mexican dry forest (Mooney *et al*., 1989).

Because of the diversity of orchids and the difficulties in obtaining live plants from the canopy for studies of nocturnal acidification, most surveys for CAM in orchids involve isotopic analysis, a technique that may not effectively identify C_3_ + CAM or facultative CAM species. C_3_ + CAM and facultative CAM orchid numbers are thus not well enumerated but could be substantial. For example, in a study of 200 species of Panamanian epiphytic orchids in which 21 % had CAM-type isotopic signatures, a further 30 % of the species had C_3_-type δ^13^C values but showed significant nocturnal tissue acidification (Silvera *et al*., 2005). Despite the potentially large numbers of orchids with C_3_ + CAM, facultative CAM might be uncommon, having been reported only in *Dimerandra emarginata* (Zotz and Tyree, 1996; Winter, 2024), a long-lived bark-epiphyte present in forests from Central America to the Atlantic forests of Brazil. It grows in exposed parts of the canopy and exhibits a C_3_-type δ^13^C value of −27.5 ‰ (Silvera *et al*., 2010).

The most basal CAM-containing orchid group is the tribe Vanilleae, a group of 189 mainly terrestrial species from the Old and New World tropics that arose ~63 Mya. The largest genus, *Vanilla*, diverged ~61 Mya, and is now a group of 115 succulent-leaved climbing vines (Cameron, 2011; Givnish *et al*., 2015). *Vanilla* is one of few orchid genera with a transoceanic distribution. A North and South American, African and Asian distribution can be explained by recent long-distance dispersal of seeds but might also be indicative of an older origin, before the complete separation of Gondwana. Of the five species of *Vanilla* for which δ^13^C values are available, *V. fragrans*, *V. pompona* and *V. planifola* exhibit CAM-type values more positive than −21 ‰, whereas *V. trigonocarpa* exhibits a C_3_ + CAM value of −21.7 ‰. C_3_-type values of −23.4 and −29.0 ‰ are reported for *V. inodora* (= *V. pfaviana*) (Zotz and Ziegler, 1997; Silvera *et al*., 2005, 2010). *Vanilla planifolia* is an unexpected candidate for the earliest known CAM species. *Welwitschia mirabilis* pre-dates *V. planifolia*, but it is uncertain when CAM appeared in *W. mirabilis*. The crown diversity of current species of *Isoëtes* is dated broadly similar to *V. planifolia* when estimated from nuclear genes, but post-dates *V. planifolia* by ~20 Mya when estimated from chloroplast genes (Givnish *et al*., 2015; Wood *et al*., 2020).

*Dendrobium*, with an Oligocene stem age of ~41 Mya, is a widespread, predominately epiphytic orchid genus of ~1686 species containing Australasian and Asian clades that diverged ~37 Mya (Li *et al*., 2019). The crown age of the Asian clade, ~31 Mya, is significantly older than the Australasian clade crown age of ~20 Mya. A survey of 97 species from the Asian and Australasian clades identified C_3_ as the ancestral photosynthetic state in *Dendrobium* (Li *et al*., 2019). CAM might have evolved independently eight times across the genus, four times in each clade. Concomitant with earlier diversification of the Asian clade, CAM appeared earlier in the Asian clade. The four Asian CAM lineages appeared between ~22 and 12 Mya and diversified between ~17 and 0.8 Mya. The origins of the Australasian CAM lineages ranged from the middle to late Miocene, between ~16 and 6 Mya, with diversification occurring ~11–4 Mya. Selection for xerophytic traits, such as CAM, might be expected during this period, at least in Australia, where forests retreated eastwards upon exposure to increased Miocene aridification. Australian *Dendrobium* are currently restricted to the wet forests and drier sclerophyllous vegetation of the eastern ranges and coastal plains, where Plio- and Pleistocene cool–dry to warm–wet climatic oscillations caused repeated contraction, expansion and isolation of the rainforests, sclerophyllous forests and open woodlands and grasslands (Byrne *et al*., 2011; Simpson *et al*., 2018).

The ancestors of Madagascar’s most species-rich orchid genus, *Bulbophyllum*, were probably C_3_. During the late Miocene, ~7 Mya, following a C_3_-to-CAM transition, a CAM *Bulbophyllum* clade from the sub-humid Central Highlands colonized and diversified in the warmer and moister Eastern Lowlands. Subsequently, elements of this lowland CAM clade occupied adjacent hotter north-west seasonally dry tropical forests (Gamisch *et al*., 2021). Although it is unclear whether CAM was a synapomorphy that enabled the unusual niche shift of the CAM clade into the high-rainfall coastal forests, CAM was probably a trait that assisted further movement into the seasonally dry forests. The radiation of the CAM clade, which now constitutes ~16 % of Madagascar’s 190 species of *Bulbophyllum*, into new ecological space increased the species richness of *Bulbophyllum* but did not affect the rate of species diversification.

Despite the rarity of CAM in terrestrial orchids, CAM has contributed to the diversification and extension of at least two groups, the Sobralieae (Silvera *et al*., 2009) and the Eulophiinae (Bone *et al*., 2015*a*, *b*). Analysis of the Eulophiinae provides an opportunity to document interactions between CAM and diversity of orchids in dryland ecosystems that expanded during the late Miocene. Nested within the Epidendroideae, a species-rich subfamily, of which 90 % of species are epiphytes (Zotz *et al*., 2021), the Eulophiniiae is a mainly terrestrial subtribe of ~270 species in nine genera that are native to the Old World tropics of Africa and Madagascar, Asia and Australasia (Bone *et al*., 2015*a*; Freudenstein & Chase, 2015). The most species-rich genera are *Eulophia*, with 60 % of the species, and *Oeceoclades*, with 19 %. Given that epiphytes are restricted to six species in four basal genera, it is inferred that the terrestrial condition arose from epiphytic ancestors (Fig. 3).

CAM evolved four times within the terrestrial Eulophiinae (Bone *et al*., 2015*b*), once at the base of the *Oeceoclades* (12–6 Mya) and three times in small clades within *Eulophia* (10, 8 and 7–4 Mya). The transition from epiphytism to terrestrialism is strongly associated with colonization of dry environments, but not with the shift from C_3_ to CAM. CAM apparently evolved subsequently in response to aridity, perhaps as a culminating trait (Males, 2016). The four CAM lineages occupy different spaces along a climatic gradient associated with dry and seasonally dry habitats (Bone *et al*., 2015*b*). *Oecoclades* are mainly restricted to seasonally dry deciduous forests. Two single-species CAM clades within *Eulophia* are *Aloë*-like in morphology (Bone *et al*., 2015*b*). *Eulophia petersii* has expanded its range, tracking aridification to south-central Africa and to the Arabian Peninsula, whereas *Eulophia leachii* is limited to low-elevation dry and riverine forest margins in southern Africa, suggesting that this species could have contracted its range or has reached the limit of its available niche. The most recent clade of *Eulophia* inhabits seasonal grasslands and dry forest margins. The five species are CAM, with succulent juvenile leaves and grass-like mature leaves.

### CAM in the Bromeliaceae

**Fig. 4.**
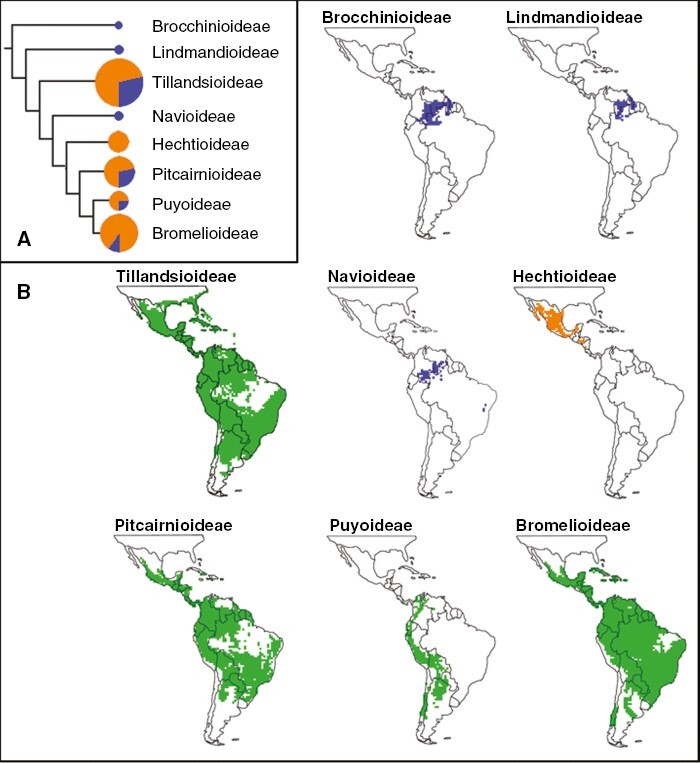
(A) Proportion of species without CAM (blue) or with CAM (orange) within the eight subfamilies of the Bromeliaceae. The size of the circle indicates the relative number of species. The figure is modified from Givnish *et al*. (2014) and Crayn *et al*. (2015). (B) Distribution of species within subfamilies of the Bromeliaceae in which CAM is absent (blue), CAM and C_3_ species are present (green), and CAM only is known (orange). The figure is modified from Zizka *et al*. (2020).

The Bromeliaceae originated as terrestrial C_3_ plants in the infertile perhumid Guayana Shield during the mid-Cretaceous, ~97 Mya (Crayn *et al*., 2004; Givnish *et al*., 2011, 2014). A long phylogenetic ‘fuse’ (*sensu*Ramírez-Barahona *et al.*, 2020), a feature common within angiosperm families of tropical humid biomes, was followed by divergence of the extant subfamilies that began ~22 Mya and accelerated between 17 and 11 Mya (Givnish *et al*., 2011, 2014). The current species-rich, structurally and geographically diverse family contains eight subfamilies, 79 genera and ~3709 species (Fig. 4; Gouda and Butcher, 2022). Nearly two-thirds of extant bromeliads belong to two large radiations: the core tillandsioids, originating in the Andes ~14 Mya, and the Brazilian Shield bromelioids, originating in the Serro do Mar and adjacent regions ~9 Mya (Givnish *et al*., 2011).

Distributed from southern USA in the north, Caribbean islands to the east, to Tierra del Fuego in the south, bromeliad diversity is centred in the Andes, Central America, the Guayana Shield, the Serra do Mar of Southeast Brazil, and the Brazilian Shield (Smith & Downs, 1979). To the west, a few species inhabit the Juan Fernandez (*Greigia berteroi* and *Ochagavia elegans*) and Galápagos Islands (*Racinaea insularis*) in the Pacific. The only bromeliad not endemic to the Neotropics, *Pitcairnia feliciana*, grows in Guinea in Western Africa, apparently the result of long-distance dispersal event ~12 Mya (Givnish *et al*., 2004).

Bromeliad expansion and diversification were triggered by major geological, climatic and atmospheric changes and facilitated by the selection and accumulation of morphological, reproductive and physiological traits that probably improved the efficiency of nutrient and CO_2_ uptake, water retention, pollination and seed dispersal (Crayn *et al*., 2004, 2015; Givnish *et al*., 2011, 2014; Males and Griffiths, 2017, 2018). The traits, which included the development of tanks (tightly overlapping rosettes of leaves that hold water), absorptive trichomes, epiphytism, neotony (retention of juvenile structure, as in the atmospheric *Tillandsia*), carnivory, CAM, avian pollination and entangled seeds, increased the ecological proficiency of bromeliads, enabling expansion into arboreal habitats and the invasion of semi-arid and arid biomes at both lowland and higher elevations.

CAM has evolved at least five times in the Bromeliaceae (Fig. 4; Crayn *et al*., 2004; Givnish *et al*., 2011, 2014). The ancestral state is presumed to be C_3_ photosynthesis, because CAM is unreported in the sister family of the Bromeliaceae, the marsh-inhabiting Typhaceae (Ramírez-Barahona *et al*., 2020; POWO, 2022). The interaction between CAM and the diversification rate of bromeliads is unclear. Schulte *et al*. (2010) reported that CAM is correlated with increased net diversification, but Givnish *et al*. (2014) observed no significant difference in diversification rates between purely CAM and C_3_ lineages.

CAM is reportedly absent from the Brocchinioideae and the Lindmanioideae, two basal subfamilies that are sister to other bromeliads (Fig. 4; Crayn *et al*., 2004, 2015; Givnish *et al*., 2011, 2014), which is perhaps not surprising considering that both tend to inhabit mid- to high-elevation perhumid low-nutrient sites. In *Brocchinia*, tanks (which appeared ~9 Mya) and capped absorptive hairs are more likely to assist nutrient absorption than to reduce water stress. Even so, in a survey of 46 of the 59 brocchinioid and lindmanioid species that generated a C_3_-type mean δ^13^C value of −25.4 ± 2.2 ‰ (Crayn *et al*., 2015), seven species had δ^13^C values of between −19.6 and −22.9 ‰, which might, in some circumstancess indicate a contribution of CAM-type dark CO_2_ uptake to whole-plant carbon gain (Winter and Holtum, 2002).

The Naviodeae, like the Lindmanioideae, remained terrestrial, non-tank species of the Guyana Shield (Fig. 4), with the exception of a monotypic genus, *Cottendorfia florida*, in Northeast Brazil (Givnish *et al*., 2014; POWO, 2022). The subfamily appears C_3_ on the basis of δ^13^C values of 27.8 ± 2.5 ‰ (mean ± s.d.) for 73 of the 113 species (Crayn *et al*., 2015).

The Hechtioideae, together with the tillandsioids, is the earliest CAM-containing bromeliad clade. *Hechtia*, the only genus, contains 86 bee-pollinated, non-tank, terrestrial species, with fleshy spiny leaves. In an isotopic survey of 28 species of *Hechtia*, all δ^13^C values were indicative of strong CAM (−13.9 ± 2.8 ‰; Fig. 4; Crayn *et al*., 2004, 2015). Neither C_3_ nor putative C_3_ + CAM species were detected.

The divergence of *Hechtia* in the mid- to late Miocene (stem node, 16 Mya; crown node, 10 Mya; Givnish *et al*., 2014), a period of increasing aridification, warm but decreasing temperatures and declining atmospheric [CO_2_], broadly coincides with the spread of aridity in Central America and expansion of arid-zone terrestrial CAM groups, such as the Agavaceae and Cactaceae (Arakaki *et al*., 2011). With a centre of diversity in semi-arid Mexico, *Hechtia* now extends from Texas in the north to Honduras and Nicaragua in the south (Benzing, 2000). It is unclear how ancestral *Hechtia* colonized Central America. They could have moved along the Panamanian land bridge from the Guyana Shield or western Andes, because the Isthmus of Panama was almost closed by the Middle Miocene (Montes *et al*., 2012; O’Dea *et al*., 2016), or long-distance dispersal is possible.

The Tillandsioideae is the largest bromelioid subfamily, with 19 genera and 1420 species (Barfuss *et al*., 2016; Gouda and Butcher, 2022). Arising probably in the northern Andes or Central America during the mid-Miocene (stem node, 17 Mya; crown node, 15 Mya; Givnish *et al*., 2011, 2014), the Tillandsioideae diverged and expanded into southern North America, the Caribbean, along the Andes, across northern South America and the Guyanan Shield down into the Brazilian Shield and into Southeast Brazil (Fig. 4; Smith and Downs, 1979; Givnish *et al*., 2011). Expansion occurred as landscapes changed extensively; the northern Andes lifted in the mid-Miocene, the Amazon shifted course, the Serra do Mar of Southeast Brazil lifted in the late Miocene, and the Isthmus of Panama closed in the late Miocene/early Pleistocene. Expansion of tropical and cloud forests, the formation of extensive, often fertile, heterogeneous cordillaras, the development of seasonally dry Central American landscapes and the creation of rain-shadows along the Andes provided habitats in which diversifying selection could occur. Long-distance movement and colonization were probably aided by avian pollination and seed dispersal (Givnish *et al*., 2011).

Most Tillandsioideae are epiphytic or lithophytic, with diversity peaking at mid-elevations in northern Peru to Columbia, especially in montane forests, cordilleras and across the Caribbean. They include shade-tolerant rainforest plants, through light-demanding species of exposed canopy sites, to xeromorphic drought-tolerant species of semi-deciduous forests and thorn woodland (Benzing, 2000). Atmospheric species that have lost tanks, absorbing water via trichomes and using roots as holdfasts, can be epiphytic or even live in sand (e.g. *Tillandsia landbeckii* Phil. from Peru and Chile).

The conclusion that CAM evolved three times within the Tillandsioids, twice in *Tillandsia* and once in a common ancestor to *Tillandsia* (Crayn *et al*., 2004; Givnish *et al*., 2014), is likely to be an underestimate. CAM-type δ^13^C values of −18.1 and −18.6 ‰ are reported for *Vriesia barclayana* and *V. espinosae* (Pierce *et al*., 2002*a*), which are now placed within *Tillandsia* (Barfuss *et al*., 2016; Gouda and Butcher, 2022). In *Lemeltonia* (formerly in *Tillandsia*), a semi-xerophytic genus from which tanks have been lost (Barfuss *et al*., 2016), *L. triglochinoides* has a CAM-type δ^13^C value of −13.9 ‰, and two other *Lemeltonia* have C_3_ + CAM values of −23.1 and −23.5 ‰ (Crayn *et al*., 2015). C_3_ + CAM-type δ^13^C values of −21.5 ‰ for the Tillandsioids *Alcantarea duarteana* and −21.3 ‰ for *Mezobromelia schimperiana* (Crayn *et al*., 2015) also warrant further investigation for CAM.

Isotopic values of the majority of the tribe Tillandsieae (*Gregbrownia*, *Guzmania*, *Pseudalcantarea*, *Barfussia*, *Wallisia*, *Racinea* and *Tillandsia*), apart from *Tillandsia*, are overwhelmingly C_3_ type, supporting a C_3_ origin for *Tillandsia*. C_3_ + CAM in *Guzmania monostachia*, which has a δ^13^C value of −25.1 ‰, and a δ^13^C value of −20.1 ‰ for *Racinaea fraseri*, indicate the presence of low-level CAM.

Within the genus *Tillandsia*, 15 of the 16 clades recognized by Barfuss *et al*. (2016), including all atmospheric tankless forms, contain plants with CAM-capable δ^13^C values (δ^13^C values more positive than −21 ‰; Crayn *et al.,* 2015). The exception is the *Tillandsia australis* complex of lithophytic mesophyte tank-containing plants, for which a single value of −24.4 ‰ is known. Two groups within the more basal *Tillandsia* clades, the xerophytic tankless *Tillandsia* subgenus *Tillandsia* and the *Tillandsia biflora* complex of mesophytic species with tanks, contain species with CAM-type and with C_3_-type isotopic values. Several species within *Tillandsia* subgenus *Pseudovriesea*, a xerophytic group without tanks, have isotopic values common in C_3_ + CAM plants. Clearly, CAM designation in these lineages requires more detailed measurements of gas exchange and nocturnal acidification by stressed and well-watered plants, such as those of Pierce *et al*. (2002*a*, *b*).

The Pitcairnioideae arose ~14 Mya near the Guayana Shield (Givnish *et al*., 2011). Among the basal genera, *Pitcairnia* appeared ~13 Mya in the northern Andes and its lowland slopes, before expanding in range and diversifying to the northern and central Andes, Guayana Shield, Central America, Amazon basin, Southeast Brazil and the Caribbean (Fig. 4). It is now the second-largest bromeliad genus, with 493 species, with an elevational range from sea level to above the tree line. Sister to *Pitcairnia*, *Fosterella* (35 species) originated ~11 Mya in the central Andes. CAM is absent from *Pitcairnia* and *Fosterella*, many of which are broad-leaved, mainly terrestrial species of rainforest and cloud-forest understories although both genera include moderately drought-tolerant members that inhabit more protected shaded and humid microhabitats within rocky landscapes (Fig. 4; Crayn *et al*., 2004, 2015). Indeed, the centre of *Fosterella* diversity includes areas of the Bolivian Andes in which seasonally dry tropical forests are considered ancestral biomes to Bromeliaceae as a whole (Givnish *et al*., 2011).

CAM arose from C_3_ photosynthesis in the Pitcairnioideae in a clade sister to *Fosterella* that arose ~11–9 Mya, possibly in the Andes of south-central Bolivia (Givnish *et al*., 2011, 2014). The *Deuterocohnia*, *Dyckia* and *Encholirium* clade contains terrestrial and lithophytic, rosette-leaved, tankless, spiney, xeric species, of which 40 % of the 232 species analysed exhibit δ^13^C values expected for strong CAM (11 of 16 *Deuterocohnia* −11.9 ± 0.9 ‰; 68 of 177 *Dyckia* 12.0 ± 1.3 ‰; and 14 of 36 *Encholirium* 12.2 ± 1.3 ‰; Crayn *et al*., 2015).

*Deuterocohnia*, which diverged ~9 Mya, are bird- and insect-pollinated cushion plants of arid, high-elevation xeric habitats in the southern Andes, northern Argentina, and south and eastern Brazil (Schütz *et al*., 2016). Its bat-pollinated sister clade, *Encholirium*, restricted to rocky outcrops/grasslands and inselbergs in arid Northeast Brazil, gave rise to *Dyckia* ~2 Mya when it invaded the Brazilian Shield from the Andes (Krapp *et al*., 2014). The hummingbird- and insect-pollinated *Dyckia*, which typically inhabit xeric infertile highly exposed rocky outcrops, cliffs, slopes and inselbergs, have a centre of diversity in mountainous regions of the central Brazilian cerrado, ranging into the adjacent Atlantic Forest and caatinga in the east, to the chacos in the west, and into Uruguay and the northern Argentinean pampas in the south (Smith and Downs, 1979; Krapp *et al*., 2014).

The Puyoideae, sister to the Bromelioideae, contains ~257 species of the genus *Puya* (POWO, 2022). All are terrestrial tankless rosette-forming bromeliads, with xerophytic features that include water-absorbing trichomes and internal leaf hydrenchyma. Typically hummingbird pollinated, *Puya* reproduce only once in their life (monocarpous), although many form colonies of attached clonal rosettes, a feature lost in some high-elevation species.

Originating in central Chile during the mid-Miocene, *Puya* (stem node, 11 Mya; crown node, 9 Mya; Givnish *et al*., 2014) radiated northwards during the late Miocene and the Pleistocene during the final uplift of the Andes (Jabaily & Sytsma, 2013; Schulte *et al*., 2010). The genus comprises a clade endemic to lowland and coastal habitats in central Chile and a more species-rich, almost exclusively Andean clade that extends as far north as Panama and Costa Rica (Fig. 4). *Puya* are found from sea level to >4500 m a.s.l. in habitats as diverse as coastal Chilean woodlands and scrub, short-statured, lowland arid forests and savannas, mesic and xeric inter-Andean valleys, high-elevation plains above the timberline and treeless windswept tundras in the higher Andes. *Puya* diversity increases towards the equator, with most species found at ~2600–3300 m a.s.l., above the moist lowland forests and below environmentally harsh high-elevation habitats. Narrow endemism and highly fragmented distributions are common, particularly in higher-elevation species (Jabaily and Sytsma, 2013).

First impressions from superimposing values from a δ^13^C survey of 132 of 257 *Puya* species (Crayn *et al*., 2015) onto the phylogeny of Jabaily and Sytsma (2013), and assuming that CAM is indicated by δ^13^C values more positive than −21 ‰, are that: (1) 27 % of *Puya* exhibit CAM (Fig. 4); (2) CAM has arisen a number of times in *Puya*; and (3) CAM is more prevalent in the older Chilean and southern Andean clades than in the northern Andean clades. Both CAM and C_3_ species are present in the southern basal, lowland ‘blue *Puya*’ clade. More definite assignations await a more extensively sampled and better-resolved phylogeny and a clearer understanding of the extent of hybridization. It is currently unclear whether C_3_ or CAM photosynthesis is the ancestral character state in Puyoideae (Crayn *et al*., 2004; Schulte *et al*., 2010; Givnish *et al*., 2014; Silvestro *et al*., 2014).

The relative abundance of CAM *Puya* taxa decreases with increasing elevation, but four species with CAM-type δ^13^C values have elevational ranges that extend above 4000 m a.s.l., and ten occur >3000 m a.s.l., clearly evidence that CAM *per se* is not incompatible with the subzero night-time temperatures that characterize these high-elevation sites (Crayn *et al*., 2015).

The Bromelioideae is the most recent and the third-most species-rich bromeliad subfamily, with ~990 species in 39 genera (Gouda and Butcher, 2022). Despite containing the agriculturally significant *Ananas comosus* (pineapple) and many species of horticultural interest, the Bromelioideae phylogeny is the least well-resolved bromeliad subfamily phylogeny, because sequence divergence is low, many morphological characters are highly homoplastic, and sampling across the genera has been uneven (Evans *et al*., 2015). For example, *Aechmea*, a genus that constitutes 25 % of the bromelioids, is highly polyphyletic, with members in 12 different lineages (Evans *et al*., 2015).

About 90 % of Bromelioideae are CAM, in comparison to 24–28 % of the Tillandsiodeae, Puyoideae and Pittcairnioideae (Fig. 4). The Bromelioideae and its sister Puyoideae probably diverged from the Pitcairnioideae ~15 Mya, with CAM arising at the base of the Bromelioideae–Puyoideae ~11 Mya (Givnish *et al*., 2014), although it is unclear whether C_3_ or CAM is the ancestral photosynthetic state of the Bromelioideae. Terrestrial and tankless lineages near the base of the bromelioid phylogeny contain C_3_ or CAM species (Schulte *et al*., 2009; Silvestro *et al*., 2014; Givnish *et al*., 2015; Evans *et al*., 2015). Consistent with an origin of Bromelioideae in the vicinity of the southern Andes, three C_3_ genera, *Fascicularia* (one species), *Ochagavia* (four species) and the Chilean *Greigia* are mostly southern Andean temperate species from low elevations, including littoral sites and the Juan Fernandez Islands. With a highly disjunct distribution, most *Greigia* species inhabit high-elevation humid cloud-forest and páramo sites along the central and northern Andes, into Mexico and across to Venezuela (Will and Zizka, 1999).

Bromelioids that dispersed west to the Brazilian Shield (stem, 9 Mya; crown, 8 Mya; Givnish *et al*., 2014), presumably traversing semi-arid habitats, include the xeromorphic, mainly terrestrial CAM genera *Deinocanthon*, *Bromelia*, *Pseudananas*, *Ananas*, *Cryptanthus* and *Orthophytum*. Later, a bromelioid epiphytic clade arose around the ranges of Southeast Brazil ~6 Mya, thus CAM apparently pre-dated epiphytism in the Bromelioideae. Epiphytism diversified and radiated in the cooler and wetter climates that accompanied the uplifting of the central Andean Altiplano and the Serra do Mar of Southeast Brazil towards the end of the Miocene and the Pliocene–Pleistocene (Givnish *et al*., 2014). The centre of diversity of the core epiphytic Bromelioideae is now the Atlantic Forest and Rio de Plato regions in Southeast Brazil.

Current phylogenies support a hypothesis of repeated instances of dispersal and subsequent diversification of bromelioids outside of Brazil and the subsequent colonization and diversification in northern South America, the Andes, and Central America and the Caribbean (Givnish *et al*., 2014). The disjunct distributions and geographical conservatism among some bromeliad clades could well reflect isolation of populations following the expansions and contractions of ranges and habitats during the drying–wetting–cooling–warming cycles of the Pliocene and the Pleistocene.

Most Bromelioideae (92 %) are in the eubromelioid clade (Evans *et al*., 2015; = the tank-epiphyte clade of Givnish *et al*., 2015 plus *Ochagavia*, = the core bromeliads of Crayn *et al*., 2004 plus *Fernseea*, *Ananas*, *Anthophytum*, *Disteganthus* and *Cryptanthus*), an overwhelmingly epiphytic, tank-containing CAM group. Two early eubromelioid genera *Fernseea* (two species) and *Acanthostachys* (40 species) are exceptions, in that they are mainly lithophytic and contain a substantial proportion of C_3_ species and species with δ^13^C values between −23 and −21 ‰. Within the main body of CAM tank epiphytic eubromelioids, *Nidularium*, *Wittrockia* and *Ronnbergia* also contain plants with C_3_-type δ^13^C values, mostly rainforest and cloud-forest species (Crayn *et al*., 2004). C_3_-type values do not necessarily indicate a reversion from CAM to C_3_ (Givnish *et al*., 2011; Silvestro *et al*., 2014; Crayn *et al*., 2004; Evans *et al*., 2015), because both C_3_ and CAM are known in *Cryptanthus*, a more basal genus.

CAM was probably not a driver of diversity across all the Bromeliaceae (Givnish *et al*., 2014), because not all CAM Bromeliaceae radiated, and even the Tillansioideae radiated initially in the absence of CAM (Crayn *et al*., 2004, 2015). Nonetheless, through repeated association with periodically water-limited sites (Givnish *et al*., 2014), CAM might be considered a component of ‘synnovation’ (‘innovation’ plus ‘synergy’; Donoghue and Sanderson, 2015), an interacting combination of traits with joint consequences for adaptation and diversification (Males, 2016). For example, the tank habit, epiphytism and CAM are so closely linked that it is difficult to gauge their individual phylogenetic effects (Givnish *et al*., 2014; Silvestro *et al*., 2014). Testing of the proposition that CAM in bromeliads is a flexible and culminating trait in a cascade of adaptations that together enable plants to colonize periodically dry sites, rather than a trait that precipitates expansion of range or diversity once evolved (Males, 2016, 2017), requires a better understanding not only of the relevant traits but also of the prevalence of C_3_ + CAM in strong-CAM-containing clades and in their currently designated ‘C_3_-clade’ ancestors (cf. Pierce *et al*., 2002*b*). It is unclear whether CAM preceded or evolved alongside epiphytism. Also, sticky and entangled seeds might have influenced the appearance, diversification and radiation of bromeliad epiphytes (Givnish *et al*., 2014).

### CAM in the Crassulaceae

Crassulaceae give the name to CAM because many early investigations of diurnal acid fluctuations in leaves were performed upon members of the family (Kluge and Ting, 1978). Originating ~55 Mya ‘outside sub-Saharan Africa’ (Bruyns *et al*., 2019), Crassulaceae is the most species-rich family within the Saxifragales and the only super-rosid family that contains CAM. All >1400 Crassulaceae are leaf-succulents, but some have succulent shoots with deciduous leaves (Thiede and Eggli, 2007). Crassulaceae are distributed mainly in the temperate and subtropical regions of the Northern Hemisphere and Africa/Madagascar, with ~900 species in Mexico and Southwest USA, the Mediterranean and Macaronesia, and in Southeast Asia/Himalayas. Taxa are less common in South America and Australia. Most inhabit semi-arid rocky habitats with seasonal precipitation. Some genera are more common in arid mountainous habitats and at higher elevations, but few species inhabit arid deserts.

The family is currently subdivided into three subfamilies (Bruyns *et al*., 2019; Messerschmid *et al*., 2020; POWO, 2022) that each contain strong-CAM and C_3_ + CAM plants: Crassuloideae (one genus), Kalanchoideae (four genera) and Sempervivoideae (30 genera; Table 1), with *Perriero-sedum* unplaced. CAM has been detected in 21 of the 36 genera. Nevertheless, C_3_ + CAM is postulated as ancestral in Crassulaceae, with at least one independent evolution of strong CAM in each subfamily (Gilman *et al*., 2023). Not all members of Crassulaceae might be capable of CAM, because δ^13^C values of many *Aichryson* are more negative than −25 ‰ (Tenhunen *et al*., 1982; Messerschmid *et al*., 2020), and Teeri (1982) observed no day–night changes in titratable acidity in *Sedum ternatum*. More surveying for low-level CAM is required.

**Table 1.** Taxonomy of the family Crassulaceae modified from Messerschmid *et al.* (2020) and distributions from Thiede and Eggli (2007). Bold indicates that CAM has been demonstrated in a genus (Gilman *et al.*, 2023). *Sedum* is now considered polyphyletic so various *Sedum* clusters are shown in different clades, followed by “in part”.

Subfamily/clade	Genus	Species	Distribution
Crassuloideae	***Crassula***	200	Mainly southern Africa, Madagascar. Some in Northeast Africa, Arabian Peninsula, Southeast Asia, Australasia, New World or cosmopolitan
Kalanchoideae	***Adromischus***, ***Cotyledon***, ***Kalanchoë***, ***Tylecodon***	240	Mainly southern Africa and Madagascar. Some in South Asia, East Asia, Southeast Asia, Philippines and Indonesia
Sempervivoideae		1040	
Telephium clade	***Hylotelephium***, *Kungia*, *Meterostachys*, ***Orostachys***, *Phedimus*	160	Mainly temperate Asia, Eastern Mediterranean
	*Pseudosedum*, *Rhodiola*, *Sinocrassula*, ***Umbilicus***		
Sempervivum clade	*Petrosedum*, ***Sempervivum***, *Jovibarba*	60	Europe, Mediterranean, Near East, Northeast Africa
Aeonium clade	***Aeonium***, ***Aichryson***, ***Monanthes***, *Hypagophytum*,	75	Mainly North Africa, Macaronesia
	***Sedum***, in part (eight species)		
Leucosedum clade	*Pistorinia*, ***Rosularia***, *Prometheum*, *Afrovivella*, *Sedella*, ***Dudleya***	200	Europe, Mediterranean, Near East, Central Asia, North America
	***Sedum***, in part (~120 species)		
Acre clade	***Cremnophila***, ***Echeveria***, ***Graptopetalum***, ***Lenophyllum***,	550	Asia, Europe, Macaronesia, North America, Central America, South America
	***Pachyphytum***, *Thompsonella*, ***Villadia***, ***Sedum***, in part (~345 species)		

Subfamily Crassuloideae is basal, monophyletic and monogeneric. The ~200 taxa in *Crassula* vary in habit from small herbs to woody shrubs (Bruyns *et al*., 2019); ~30 are aquatic or semi-aquatic (Eggli, 2003). *Crassula* probably originated as perennial terrestrials in the semi-arid winter-rainfall Greater Cape Floristic Region (GCFR) at the tip of southern Africa ~46 Mya (Bruyns *et al.*, 2019). Succulent annuals subsequently developed independently at least six times, tending to cluster in early-diverging lineages. The third-most species-rich genus in the semi-arid Succulent Karoo Biome and the 15th largest in the mesic Core Cape Subregion, ~20 *Crassula* species grow in East and Northeast Africa, the Arabian Peninsula, Southeast Asia, Australasia and the New World. In Australia and New Zealand, the area with the greatest number of species outside Africa, most are ephemeral terrestrial annuals or cosmopolitan small aquatics. Facultative CAM has been demonstrated in the small Australian terrestrial species, *Crassula sieberiana* (Brulfert *et al*., 1991; Winter and Holtum, 2017).

The three major clades of *Crassula* are a GCFR clade of annuals and tuberous geophytes that originated ~39 Mya (clade A), an African/Madagascan/cosmopolitan clade (clade B) appearing ~43 Mya, and a species-rich mainly perennial GCFR clade restricted to Africa (clade C) (Bruyns *et al*., 2019). Clade C originated ~37 Mya. A subclade with a compact growth form of highly succulent leaves with reduced stems but no tubers underwent pronounced radiation and diversification across southern Africa, particularly during the last 10 Myr as climate transitioned towards drier, winter‐rainfall conditions (Bruyns *et al*., 2019; Lu *et al*., 2022).

During the last 10–20 Myr, clade B has twice reached the New World and Australasia from Africa and twice reached Europe and Asia (Bruyns *et al*., 2019). *Crassula* in Madagascar, such as *C. humbertii*, arose <5 Mya and probably reached Madagascar by long-distance dispersal from Africa. Likewise, species in Eeast and Northeast Africa and on the Arabian Peninsula all arose within the last 5 Myr but are largely confined to cool, montane habitats. The only cosmopolitan *Crassula* are small, often minutely leaved aquatic annuals or small mat-forming perennials in clade B (e.g. *Crassula helmsii*).

The evolutionary implications within *Crassula* of potential contributions of CAM to the successful colonization of southern African and central American habitats that are geologically and topographically complex require further assessment. For example, the extent or lability of CAM in seasonally dry southern African *Crassula* annuals or geophytes is unclear. Fradera**‐**Soler *et al*. (2021) concluded that aridity might have influenced the evolution of leaf morpho-anatomical traits, with mesophyll traits being linked to water storage and CAM performance, but Lu *et al*. (2022) did not even address CAM as a factor that linked life form to a major shift in diversification rate in *Crassula*.

The monophyletic subfamily Kalanchoideae contains ~262 species in four genera (*Adromischus* 29 species, *Cotyledon* 18 species, *Kalanchoë* 165 species and *Tylecodon* 50 species; POWO, 2022). Arising ~23 Mya (Bruyns *et al*., 2019), distribution is now centred in southern Africa and Madagascar, but *Kalanchoë* and *Cotyledon* range to eastern Africa and the Arabian Peninsula, with *Kalanchoë* extending further into tropical parts of western and Sub-Saharan Africa and into South, East and Southeast Asia and Indonesia. In southern Africa, *Tylecodon* and *Adromischus* grow predominantly in winter-rainfall areas, *Kalanchoë* in summer-rainfall areas, whereas *Cotyledon* is distributed in both regions (Thiede and Eggli, 2007).

*Kalanchoë*, the largest and most widely distributed genus in the Kalanchoideae, arose in humid habitats in Madagascar, from which it radiated into more arid areas and thence to arid regions in eastern Africa (Gehrig *et al.*, 2001). The genus, which includes terrestrial herbaceaous leaf succulents, thin-leaved plants, tall xeromorphic perennial bushes, epiphytes and climbers (Kluge and Brulfert, 1996), many of which can propagate vegetatively (Smith *et al*., 2022), comprises three major clades (Bruyns *et al*., 2019; Messerschmid *et al*., 2020). In the ancestral Kitchingia clade, thin-leaved plants endemic to humid sites in Madagascar, carbon gain is mainly via C_3_ photosynthesis, although they may have the potential to perform some CAM when stressed (Kluge *et al*., 1991, 1993, 1995; Kluge and Brulfert, 1996; Winter, 2019). The Bryophyllum clade, also Madagascan, occurs in dry habitats with relatively predictable wet and dry seasons. Obligate CAM plants, they can supplement nocturnal CO_2_ uptake with diurnal CO_2_ uptake if sufficient water is available. Species in the Eukalanchoë clade have strongly succulent leaves and overwhelmingly perform CAM, independent of watering. Eukalanchoë species are abundant mainly in the extremely dry south of Madagascar and in arid sites of eastern Africa.

CAM evolution within subfamily Sempervivoideae is difficult to assess because the largest genus *Sedum* is paraphyletic across three clades containing 18 genera (Table 1). Rearrangement could result in the subfamily containing between 30 and 13 genera (Messerschmid *et al*., 2020)! In what is now *Sedum*, δ^13^C values between −13.1 and −31.1 ‰ are known (Teeri, 1982; Pilon-Smits *et al*., 1996; Messerschmid *et al*., 2021). Facultative CAM is present in *Sedum acre* (Kluge, 1977), *S. album* (Castillo, 1996), *S. pulchellum* (Smith and Eickmeier, 1983), *S. sexangulare* (Schuber and Kluge, 1981) and *S. telephium* (Lee and Griffiths, 1987).

Unlike tropical/subtropical Crassuloideae and Kalanchoideae, the predominantly Northern Hemisphere temperate Sempervivoideae extended their range into the New World (Table 1; Thiede and Eggli, 2007). In general, the northern temperate clades are poor in species, whereas northern American and southern African lineages are more diverse. The ancestrally European distribution of *Sempervivum* was expanded by one long-distance dispersal event into northern Africa and three long-distance dispersal events into Southwest Asia. *Sempervivum* and *Jovibarba* are monophyletic sister genera, which split ~5–9 Mya, contemporary with the major uplift of the European alpine system (Klein and Kadereit, 2015).

### CAM in Euphorbia (Euphorbiaceae)

In the Euphorbiaceae, a family of ~8300 species, CAM is a major contributor to net carbon gain in perhaps 850 of the ~2300 species of the largest genus, *Euphorbia* (Horn *et al*., 2014), and a very small contributor to net carbon gain in *Jatropha curcas* and *J. dioica* (Winter and Holtum, 2015). C_4_ photosynthesis has also evolved in the *Euphorbia* once, in section *Anisophyllum*. The CAM *Euphorbia* lineages, which are stem-succulents with swollen photosynthetic branches, thin bark and ephemeral reduced leaves, were ancestrally woody, whereas the C_4_-containing *Anisophyllum* arose from ancestrally leafy herbaceous ancestors (Horn *et al*., 2012).

The proportions of succulents and CAM differ among the four major lineages of *Euphorbia*. Subgenera *Euphorbia* and *Rhizanthium* are overwhelmingly succulent, exclusively perennial and contain many CAM taxa. In contrast to these crown clades, some basal groups lack photosynthetic stems and have well-developed, albeit ephemeral leaves. Subgenera *Chamaesyce* and *Esula* contain a few ephemeral-leaved, green-stemmed CAM succulents nested among mainly non-succulent leafy C_3_ and C_4_ shrubs and annuals.

*Euphorbia* diversified after Africa, South America, Madagascar and India detached from Gondwana (95% highest posterior density age estimates: crown clade, 41–55 Mya; stem clade, 48–62 Mya), with CAM evolving independently 16–21 times, principally from the Miocene onwards (Horn *et al*., 2014). With a nearly global distribution, *Euphorbia* are currently most abundant in warm, seasonally dry and arid ecosystems of the tropics of Africa, Macaronesia, Madagascar, Eurasia, the New World and, to a lesser extent, Australia.

The δ^13^C values of non-C_4_*Euphorbia* (Horn *et al*., 2014) show a marked bimodal distribution, with 60 % exhibiting C_3_-type values of more negative than −23 ‰, 34% with CAM-type values more positive than −21 ‰, and 5 % with C_3_ + CAM values of between −21 and −23 ‰ (Horn *et al*., 2014). C_3_ photosynthesis is ostensibly ancestral, but insufficient data are available to assess whether C_3_ + CAM is present at the bases of the lineages with strong CAM. The 5 % of δ^13^C values between −21 and −23 ‰ (Winter, 1979; Horn *et al*., 2014), which could indicate C_3_ + CAM or facultative CAM, are not noticeably clumped in the basal regions of lineages, but without more extensive surveys of gas exchange and titratable acidities in appropriate taxa, the possibility that taxa with C_3_-type isotopic values exhibit C_3_ + CAM or even facultative CAM, as shown in *Euphorbia aphylla* (Mies *et al*., 1996), cannot be excluded. The frequency of evolutionary transitions to strong-CAM expression in *Euphorbia*, with multiple origins within each of the four subgenera, would be consistent with the pre-existence of C_3_ + CAM (i.e. would indicate fewer independent origins) or at least a proclivity for phenotypic plasticity.

Evolutionary lability is a feature of *Euphorbia* which, in addition to displaying an array of carbon-capture mechanisms, exhibit a multiplicity of growth forms. From a woody, non-succulent ancestor, there have been at least five origins of the herbaceous habit, seven transitions from herbs to secondary woodiness, and 14 origins of strongly xeromorphic growth forms (Horn *et al*., 2014). The evolution of markedly xeromorphic growth forms is associated with transitions from monopodial architecture to the sympodial architecture that is characteristic of many candelabra-shaped euphorb succulents. Within section *Euphorbia*, there is a marked convergence in form with many cacti. An important difference is that in cacti, leaves have evolved into non-photosynthetic spines, whereas many cactiform *Euphorbia* maintain an ability to form leaves, especially following rainfall. The nature of photosynthesis in these leaves, which are often deciduous, is essentially unknown.

Many clades within *Euphorbia* have subclades that inhabit widely separated regions, often different continents, yet the continental distribution of most CAM lineages in *Euphorbia* mirrors evolution *in situ* (Horn *et al*., 2014). Evolutionary access to the expression of strong CAM in *Euphorbia* might exceed the ability of CAM lineages to disperse and establish away from their continent of origin. An exception would be a CAM dispersal event in which spiny succulent species of section *Euphorbia* reached peninsular India and Southeast Asia after a single dispersal event from Africa.

CAM is associated with increased diversification in some *Euphorbia* clades (Horn *et al*., 2014). Of eight lineages that exhibited bursts of diversification between 20 and 3 Mya, a period when dryland ecosystems were expanding and the atmospheric [CO_2_] was decreasing, five were Old World monopodial stem-succulent CAM clades with lateral inflorescences, and one was the C_4_ lineage.

Most Euphorbiaceae, and even *Euphorbia*, appear to lack CAM. However, because the size and global distribution of the lineage make fine sampling difficult and because δ^13^C analysis is an imperfect CAM assessment tool, it is probable that CAM, particularly C_3_ + CAM and facultative CAM, are more common in the Euphorbiaceae than current evidence suggests.

### CAM in the Aizoaceae

The 2237 species in the five subfamilies of Aizoaceae are overwhelmingly leaf succulents, with succulence most highly developed in the Ruschioideae and Mesembryanthemoideae, and less so in the Acrosanthoideae, Aizooideae and Sesuvioideae.

The family is most diverse in the arid regions of southern Africa, with satellite centres of speciation in Australia, the west coast of South America and the Horn of Africa (Klak *et al*., 2003, 2017*a*). The Ruschioideae (1968 species), Mesembryanthemoideae (106 species) and Acrosanthoideae (seven species) are overwhelmingly South African, whereas the Aizooideae (104 species) and Sesuvioideae (52 species) inhabit mediterranean and subtropical regions in Southern Africa, North Africa, Eurasia, Australasia and South America.

**Fig. 5.**
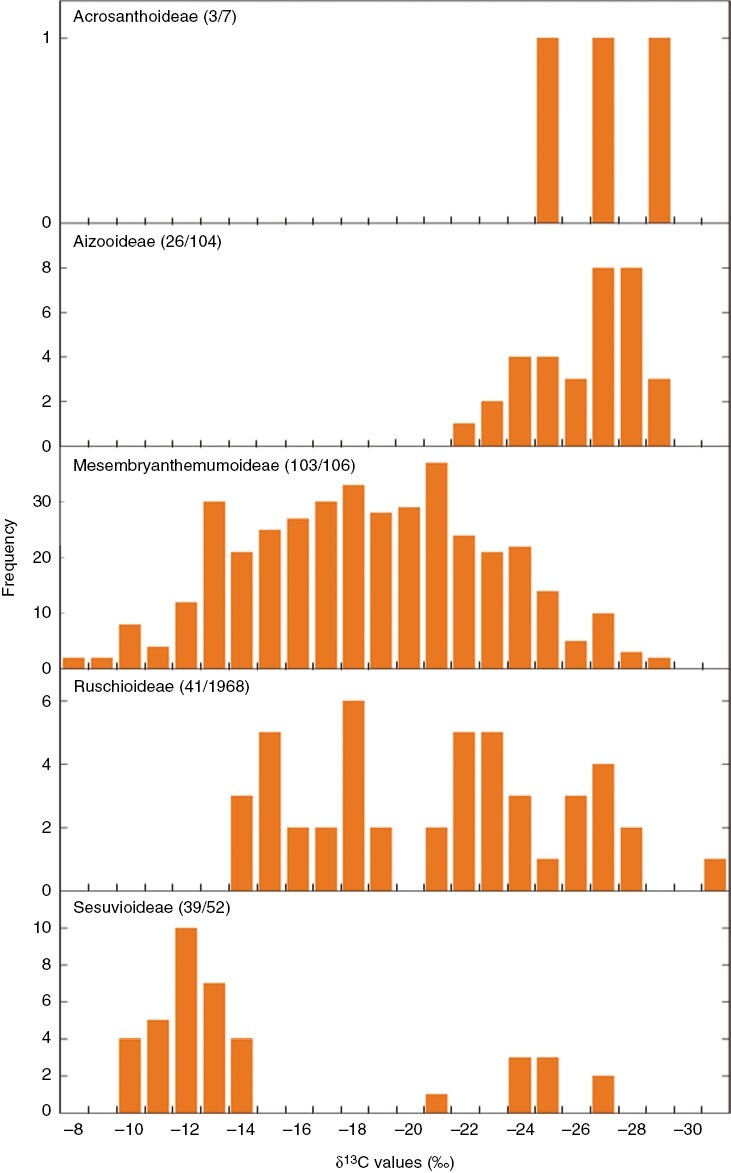
Values of δ^13^C (per mille) of taxa from the five subfamilies of the Aizoaceae. Note that frequency values include values from different species and multiple measurements of the same species. Numbers in parentheses are the number of species measured/number of species in the subfamily. Data are from Winter (2019) and Messerschmid *et al*. (2021).

Recent rapid radiations within the Aizoaceae have resulted in many taxa with few nucleotide differences between them (Klak *et al*., 2003, 2004), confounding phylogeny construction (Klak and Bruyns, 2013; Klak *et al*., 2017*a*, *b*). Denser isotopic and titratable acidity sampling has improved knowledge of CAM within the family (Winter, 2019; Winter *et al*., 2019*a*, 2021*b*; Messerschmid *et al*., 2021), providing pointers indicating where further investigation is necessary (Fig. 5).

The Aizoaceae date to ~48 Mya (Arakaki *et al*., 2011; Klak *et al*., 2017*a*). The major lineages arose in Africa between the end of the Eocene and the Oligocene, with stem dates of ~36 Mya for Acrosanthoideae, ~35 Mya for Azooideae and ~30 Mya for Sesuvioideae, i.e. essentially post-Gondwanan. The Mesembryanthemumoideae and species-rich Ruschioideae diverged ~29 Mya. The Aizooideae subsequently split into an African clade (~22 Mya) and a Eurasian–Southern Hemisphere clade (~28 Mya). As with many other Angiosperm lineages the aizoid clades have long stems with recent crown radiations. The core Ruschioideae underwent an extremely rapid major radiation ~9–4 Mya (Klak *et al*., 2004), probably post-dating the winter-rainfall/summer-arid climate that developed in south-western southern Africa ~10–15 Mya. The Mesembryanthemumoideae increased diversity at about the same time as the Ruschioideae, but with only a fraction of the species increase.

Given that the origins of the Aizoacaeae are post-Gondwanan and African, the occurrence of clades such as the Sesuvioideae in Australia and the New World required long-distance and trans-oceanic dispersal (Bohley *et al.*, 2015). The ancestors of *Gunniopsis* and *Tetragonia* apparently dispersed from Eurasia to Australasia rather than from southern Africa (Klak *et al*., 2017*a*). For *Tetragonia*, subsequent independent dispersals to South America and to southern Africa occurred in the early Miocene. For salt-adapted, coastal species with trans-oceanic distributions, such as *Sesuvium portulacastrum*, *Tetragonia tetragonoides* and *T. decumbens*, rafting or birds are the most likely vectors. As in the succulent Didiereaceae (Arakaki *et al.*, 2011; Bruyns *et al.*, 2014) and *Euphorbia* (Bruyns *et al*., 2011), within-Africa disjunct distributions occur in Aizoaceae, particularly between Southern Africa and the Horn. Such disjunctions have been interpreted variously as Pleistocene relicts, results of recent long-distance dispersals and the remnants of older arid floras and arid corridors.

CAM, especially facultative CAM, appears common in the species-rich Ruschioideae (Fig. 4). Winter (2019) detected CAM-type nocturnal malate accumulation in 43 of 48 species. Consistent with facultative CAM, 28 species showed nocturnal malate accumulation in conditions of drought and/or drought plus salinity stress, but not when well watered. In the 17 species in which significant nocturnal malate accumulation was already present in well-watered plants, nocturnal acidification was enhanced upon drought and salinity stress, consistent with a facultative-CAM component in addition to constitutive CAM. Facultative CAM could well be present in >1000 species of Ruschioideae (Fig. 5).

The spectacular recent radiation of the Ruschioideae as southern African climates changed has been attributed to their possession of highly succulent leaves with triangular cross-sections, water-stress-resisting wide-band tracheids, and seed capsules that open when wet (Klak *et al*., 2003, 2004). On the basis of δ^13^C values and acidity measurements, facultative CAM might also be associated with the expansion (Fig. 5). A phylogenetic analysis of CAM in the Aizoaceae awaits more intensive and targeted sampling of the Ruschioideae.

The Mesembryanthemoideae circumscribe only *Mesembryanthemum*, with its ~106 species of annuals, perennials and geophytes, leaf- and stem-succulents, evergreen and deciduous species, compact shrubs and woody shrubs that may exceed 1 m (Klak *et al*., 2007; Klak and Bruyns 2013). The overwhelming majority inhabit seasonally dry, winter-rainfall southern African landscapes. A few weedy salt-tolerant annuals, such as *Mesembryanthemum crystallinum* and *M. nodiflorum*, exhibit circum-Mediterranean and Arabian Peninsula distributions and have become globally distributed coastal weeds.

CAM is well known in *Mesembryanthemum*, with facultative CAM first reported in *M. crystallinum* (Winter and von Willert, 1972). Subsequently, transformation from C_3_ to CAM in *M. crystallinum* in natural conditions was demonstrated (Winter *et al*., 1978) and shown unequivocally to be under environmental control (Winter and Holtum, 2007).

In a metanalysis of 103 *Mesembryanthemum* species, including multiple samples of some species, δ^13^C values ranged from a strong CAM or C_4_-type value of ~−8 ‰ to a C_3_-type value of −30 ‰ (Fig. 5; Messerschmid *et al*., 2021). Assuming that the mean of ~−20 ‰ is not a sampling issue and assuming no C_4_*Mesembryanthemum* species, one can conclude that the plants assayed obtained anywhere between ~0 and 100 % of their carbon at night, with most obtaining ~50 % at night throughout the life of the tissue sampled. Without measurements of gas exchange or dawn–dusk tissue titratable acidities, it is unclear how many *Mesembryanthemum* are constitutively CAM, facultatively CAM or indeed whether any species lack an ability to express CAM. A key observation is that during the life cycle of *M. crystallinum* in Israel, leaf δ^13^C values change from −27 to −15 ‰ as the landscape dries and the main source of carbon shifts from daytime CO_2_ uptake to night-time CO_2_ uptake (Winter *et al*., 1978).

The Sesuvioideae probably evolved in Africa/Saudi Arabia but now occur mainly in typically hot subtropical regions of Australia and Africa, with some species in the New World and Asia (Bohley *et al*., 2015; Klak *et al*., 2017*a*). Often prostrate herbs and occasionally woody shrubs, with mildly succulent or fleshy leaves, plants may be annual or perennial, with many growing on saline or disturbed soils. The common ancestor of *Sesuvium* dispersed to North and Central America, and subsequently, the lineage repeatedly reached South America. Direct dispersal from Africa/Arabia to Australia occurred three times: once within *Zaleya* and twice within *Trianthema*. The latter lineage also dispersed to South America. East Asian regions were colonized only by *Sesuvium portulacastrum* and *Trianthema portulacastrum*.

The Sesuvioideae are the only azoid clade containing C_4_ plants. C_4_ evolved perhaps six times, in North American *Sesuvium* (formerly *Cypselea*), African *Sesuvium*, *Zaleya* and three times in *Trianthema* (Bohley *et al*., 2015). Nevertheless, only nine C_4_ sesuvioid species are known to date. Low-level CAM is present in both stems and leaves of the C_4_*T. portulacastrum*, a mostly annual, pantropical, salt-tolerant, often weedy, prostrate species with mildly succulent leaves and fleshy stems (Winter *et al*., 2021*b*). Facultative CAM, albeit at a very low level, is present in the otherwise C_3_ succulent-leaved pantropical coastal perennial *S. portulacastrum* (Winter *et al*., 2019*a*), but is as yet unreported in C_4_ members of the genus.

CAM is unreported in the Acrosanthoideae (Fig. 5; Messerschmid *et al*., 2021), a small subfamily of only seven leafy species that are endemic to the mesic fynbos in the Western Cape of South Africa. Sister to the Mesembryanthemoideae and Ruschioideae, the Acrosanthoideae diverged ~36 Mya (Klak *et al*., 2017*a*, *b*). Crown radiation during the Pliocene (±5 Mya) coincides with the expansion of the Ruschieae from the more arid karroid vegetation into the fynbos.

The diverse Aizooideae are slightly succulent to fleshy leaved. Annuals, perennials or geophytes, they may be prostrate or erect herbs to large shrubs. Ancestral to southern Africa, they are most species rich in the Karoo, although ~30 % of species are endemic to Australasia, Eurasia and South America (Klak *et al*., 2017*b*). Among the Aizooideae, CAM has been reported in two African *Tetragonia*, but not in *Tetragonia* from Australia or the New World. CAM-type acidification occurs in *Tetragonia fruticosa* (Schütte *et al*., 1967), and δ^13^C values for *Tetragonia reduplicata* from Namibian coastal and inland sites were −24.3 and −12.5 ‰, respectively (Fig. 4; Mooney *et al*., 1977). The latter value could indicate strong CAM, although, bearing in mind the presence of C_4_ photosynthesis in the Sesuvioideae, it could also be an indicator of C_4_ photosynthesis. One might expect more evidence of CAM in fleshy-leaved plants that radiated during the late Miocene/Pliocene in the succulent Karoo, with its mediterranean-like climate of low but predictable, mainly winter rainfall, seasonal droughts and ocean-influenced temperatures and fogs. Ripley *et al*. (2013) suggested that the intermittent use of C_3_ photosynthesis interspersed with periods of no positive carbon assimilation could be a successful alternative strategy to CAM for succulent taxa, such as many Aizoaceae, that contain substantial hydrenchyma in their leaves. Radiations of the most species-rich Aizooideae in southern Africa, *Tetragonia* and *Galenia*, are contemporaneous with, but much smaller than, the diversification of the sympatric Ruschioideae.

### CAM in the Portulacineae

**Fig. 6.**
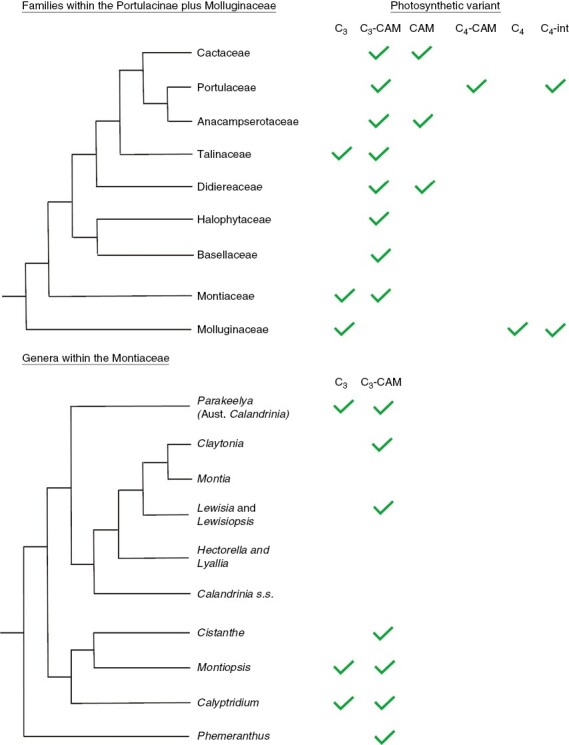
Presence of C_3_, C_3_ + CAM, strong CAM, C_4_ + CAM, C_4_ and C_4_ intermediate photosynthesis (indicated by green ticks) superimposed upon phylogenies of the Portulacineae plus Molluginaceae (upper panel) and the Montiaceae (lower panel). Absence of a symbol indicates no reports. C_3_ + CAM in the Portulacaceae is assumed for stems that do not exhibit Kranz anatomy. The phylogeny of the Portulacineae plus Molluginaceae is adapted from Ocampo and Columbus (2010), Wang *et al*. (2019) and POWO (2022). The phylogeny of Montiaceae is an amalgum of that of Hancock *et al*. (2018) and POWO (2022).

CAM is present in all eight families of the suborder Portulacineae (Caryophyllales), namely Basellaceae, Didiereaceae, Halophytaceae, Montiaceae and the ACPT clade (Anacampserotaceae, Cactaceae, Portulacaceae and Talinaceae) (Nyffeler & Eggli, 2010; Hernández-Ledesma *et al*., 2015) but appears absent from its sister group, the Molluginaceae (Fig. 6). Given that Portulacineae plus Molluginaceae diverged ~55–53 Mya and the Molluginaceae subsequently separated ~44–21 Mya (Arakaki *et al*., 2011), the origins of the Portulacineae in the New World (Ocampo and Columbus, 2010) post-date both the separation of South America and Africa between ~84 and 106 Mya and the separation of South America and Antarctica ~45 Mya (van den Ende *et al*., 2017).

In Montiaceae, the basal family in Portulacineae (Nyffeler and Eggli, 2010; Ocampo and Columbus, 2010; Ogburn and Edwards, 2015; Wang *et al*., 2019), C_3_ + CAM, often with a facultative-CAM component, is known in *Phemeranthus* (Harris and Martin, 1991), *Lewisia* (Guralnick and Jackson, 2001), *Calyptridium* (Guralnick and Jackson, 2001), *Claytonia* (Guralnick and Jackson, 2001), *Cistanthe* (Arroyo *et al*., 1990; Holtum *et al*., 2021) and the Australian *Calandrinia* (= *Parakeelya* or *Rumicastra*; see Thiele *et al*., 2018; Winter *et al*., 1981; Winter and Holtum, 2011; Holtum *et al*., 2017*a*; Hancock *et al*., 2018, 2019; Fig. 6). CAM is undetected or unreported in *Calandrinia s.s.* (= New World *Calandrinia*), *Erocallis*, *Hectorella*, *Lenzia*, *Lewisiopsis*, *Lyallia*, *Montia*, *Montiopsis* or *Schreiteria*.

The widespread herbaceous Montiaceae include succulent-leaved annuals, thick-rooted, minimally stemmed rosette perennials, stem-succulent shrubs, cushion plants and aquatic herbs (Nyffeler *et al*., 2008). Probably originating in North America, they dispersed at least twice to South America (Ocampo and Columbus, 2010, 2012), where today most *Cistanthe* and *Montiopsis* are found. *Claytonia* and *Montia* (Montieae), two genera of moist soils, most diverse at higher elevations in western America, also have wide sub-Arctic distributions and are found in northern Europe, eastern Russia, Central and South America, Australia and New Zealand. *Montia fontana* ranges from the sub-Arctic circle to islands in the sub-Antarctic (Scott, 1989). *Lewisia* mainly grow in western North America and Canada to northwestern Mexico. The New World *Calandrinia* are mainly South American, with four species extending into Mexico and North America and one endemic to the Galápagos Islands. The Australian *Calandrinia* most probably entered Australia from South America close to the estimated Australia/Antarctica final separation at ~33 Mya (Crisp and Cook, 2013; Hancock *et al*., 2018). It is unclear whether the monotypic sister genera *Hectorella* and *Lyallia*, endemic to the South Island of New Zealand and the sub-Antarctic Kerguelen Islands, respectively, are the products of long-distance dispersal from South America or from Australia (Applequist *et al*., 2006; Wagstaff and Hennion, 2007).

The dispersal of the Montiaceae across temperature, rainfall and elevational gradients has been linked to a lability in life-history strategy relative to other Portulacineae. Ogburn and Edwards (2015) argued that a herbaceous lifestyle facilitated evolutionary flexibility in the allocation of biomass to above- or below-ground organs, permitting a switch between annual and perennial life histories and enabling the exploitation of ecological opportunities following climatic and geological change. Most of the species in which CAM has been detected seem to be species of more exposed areas, often sandy or rocky.

With 71 named species, the Australian clade of *Calandrinia* is the most species-rich lineage in the Montiaceae and is most diverse genus with CAM in Australia (Winter *et al*., 1981; Holtum *et al*., 2016; Hancock *et al*., 2018, 2019). The most recent common ancestor in the lineage, most probably sister to the CAM-containing Montieae (*Lewisia* + *Montia* + *Claytonia* + *Lewisiopsis*) and the North American *Calandrinia* (of unknown CAM expression), was probably C_3_ + CAM. CAM expression within the Australian *Calandrinia* appears evolutionarily labile, with facultative CAM possibly evolving multiple times and perhaps reversions to C_3_ photosynthesis also occurring (Hancock *et al*., 2019).

The Australian *Calandrinia*, small, annual herbs or seasonally deciduous geophytes, are likely to have originated in the mesic, temperate climates of Western Australia, dispersing eastwards, southwards and northwards (Hancock *et al*., 2018). Now most species-rich along the coastal fringes of Western/south-west Australia and the semi-arid and arid regions of Central Australia, Australian *Calandrinia* also inhabit savannas and savanna–woodlands in the summer-rainfall tropics of northern Australia (into which they expanded ~18 Mya), the winter-rainfall mediterranean regions of southern Australia and southern temperate areas, including northern Tasmania and the Bass Strait islands. They are absent from rainforest regions and, in contrast to their New World relatives, from higher elevations (Hancock *et al*., 2018, 2019).

The Australian *Calandrinia* speciated and dispersed during the early to mid-Miocene, ~20–10 Mya (Hancock *et al*., 2018), when the Australian climate was generally wet, warm and stable (Martin, 2006; Byrne *et al*., 2011). Palaeo-drainage flows became irregular and seasonal lakes started to disappear, but drying out did not become widely established until the mid- to late Miocene, ~13–6 Mya (Crisp and Cook, 2013; Martin, 2006; Byrne *et al*., 2008, 2011). Lineage accumulation, pronounced during the early to mid-Miocene, underwent a sharp decline at ~10 Mya (Hancock *et al*., 2018), apparently ceasing as Australia progressively dried, with rainforests retreating to the east and open forests and woodlands spreading in the inland areas (Crisp and Cook, 2013). The early Pliocene was slightly wetter and warmer than the late Miocene, but expansion of the poles and the glacial and interglacial climate oscillations of the Pleistocene heralded severe aridity. Stony deserts formed across western and central regions of the continent, and the northern tropics became drier and more seasonal (Fujioka *et al*., 2005, 2009; Byrne *et al*., 2008, 2011). A decline in diversification rates has also been reported for other Australian plant lineages that diversified and radiated during the mid-Miocene (Crisp and Cook, 2013; Byrne *et al*., 2008). Many of these lineages appeared to respond to drying out of landscapes by retreating to moister refugia. It could be argued that the current habitats of Australian *Calandrinia* are like refugia. Irrespective of the ecosystem they inhabit, these small, ephemeral, succulent-leaved plants tend to inhabit nutrient-poor fringe environments, where water supply is ephemeral and competition from other species is low. They commonly grow in sandy or gravelly soils, intermittent watercourses and run-off areas, rock seepage lines, clay-pans and their fringes, skeletal soils on rocky hillsides, coastal or inland dunes, and saline soils.

The apparent lack of topological divergence in the *Calandrinia* phylogeny since the late Miocene–early Pliocene aridification presumably reflects continuance of some species in the face of climate-induced reduced speciation and increased extinction. The occupation by *Calandrinia* of nutrient-poor habitats with unpredictable seasonal rainfall, coupled with traits such as small size, rosette-like clusters of fleshy leaves at the base of the plant from which stems grow (often indeterminately), and C_3_ + CAM photosynthesis, enabled their establishment as annuals or annually deciduous geophytes and did not successfully select for a larger perennial, water-storing, strong-CAM habit and life cycle. It might be that the small, short lifespan, C_3_ + CAM (with/without facultative component), shade-averse phenotype has so well adapted plants to low-nutrient, water-ephemeral, rapidly-drying habitats that *Calandrinia* remained in such locations as climate changed (Hancock *et al*., 2019).

Two monotypic southern genera of the Montiaceae, *Hectorella* and *Lyallia*, probably shared a common ancestor during the late Tertiary, after the fragmentation of Gondwana (Applequist *et al*., 2006; Wagstaff and Hennion, 2007). CAM is not known in either genus but would be worth testing for, considering the frequency of CAM evolution in surrounding lineages, the presence of CAM in other taxa from high latitudes, and the ability of even low-level CAM to prolong life by reducing water loss and respiratory carbon loss during seasonal stress. *Lyallia* inhabited Kerguelen during the Pleistocene and might be a relict of an otherwise extinct Tertiary flora of the now-submerged sub-Antarctic Kerguelenian Plateau. These slow-growing cushion plants of exposed windy slopes ostensibly grow in moist environments but are often exposed to water stress because water frozen in the soil can be unavailable to plants for considerable periods (Wagstaff and Hennion, 2007). The cushion habit is an adaptation that can provide protection from low temperatures and vapour loss associated with the windy environments in which both species live.

The Basellaceae include four genera of herbaceous perennial vines with tuberous roots and slightly fleshy leaves. Most inhabit open habitats, such as scrubs, rocky slopes and sandy areas that are subject to periodic water stress. *Tournonia* (one species) and *Ullucus* (one species) are restricted to the high Andes, growing to 3500 m a.s.l. (Eriksson, 2007). *Anredera* (12 species) includes highland and lowland species native to the tropics and subtropics of the Americas and the Caribbean, whereas *Basella* (five species) is native to south-eastern Africa, Madagascar and, possibly, Asia (Anton *et al*., 2014). The centre of origin of the family is north-west South America, but a Southeast African origin is possible (Anton *et al*., 2014). Facultative CAM is present in leaves of *Anredera baselloides* (Holtum *et al*., 2018) and constitutive CAM with a facultative component in leaves and stems of *Basella alba* (Sikolia *et al*., 2009; K. Winter, Smithsonian Tropical Research Institute, unpublished observations).

The single species in the Halophytaceae, *Halophytum ameghinoi*, is an annual with succulent leaves. Endemic to the arid and semi-arid Argentine Monte region, where it grows from sea level to 2200 m a.s.l. on bare soil and in open scrubland, *Halophytum* has not been assessed for CAM, but δ^13^C values of −18.6 ‰ (Gilman *et al*., 2023) and −24.8 ‰ (Ocampo and Columbus, 2010) are consistent with variable CAM expression.

The Didiereaceae originated ~15–30 Mya. Twenty-three species are divided between three subfamilies: the Portulacarioideae (seven species) of Angola, the margins of the Namib and southern Africa, the Calyptrothecoideae (two species) of north-east and east tropical Africa, and the Didiereoideae (14 species) of Angola, Namibia, South Africa, tropical north-east Africa and Madagascar. The subfamilies arose ~12 Mya, and the genera diversified in relatively recent times, ~2 Mya (Ocampo & Columbus, 2010; Arakaki *et al*., 2011). All are perennial shrubs or tree-like, with stems that are woody and generally succulent to some extent. Leaves are succulent or fleshy and partly deciduous in the Portulacarioideae but deciduous in the Calyptrothecoideae and Didiereoideae (Bruyns *et al*., 2014). C_3_ + CAM, facultative CAM and strong CAM have been demonstrated in the Portulacarioideae and strong CAM in the Didiereoideae (Ting and Hanscomb, 1977; Winter, 1979). CAM is probably present in the Calyptrothecoideae (Sikolia *et al*., 2009).

Talinaceae (*Amphipetalum*, one species; *Talinum*, 27 species) is basal to the ACPT clade of the Portulacineae, with a most recent common ancestor age of ~9 Mya (Applequist *et al.*, 2006; Nyffeler and Eggli, 2010; Ocampo and Columbus, 2010). The family probably had its origin in South America. The African taxa form a clade, suggesting a single dispersal event to the continent. Constitutive and facultative CAM are known in *Talinum* (Kluge and Ting, 1978; Martin and Zee, 1983; Harris and Martin, 1991; Herrera *et al*., 1991; Guralnick and Jackson, 2001; Winter and Holtum, 2014; Brilhaus *et al*., 2016), a group of small perennial herbs and small shrubs with slightly succulent leaves, often with tuberous roots. Plants of seasonally mesic sites, semi-deciduous to deciduous forests and scrub, *Talinum* have colonized tropical regions of the New and Old Worlds, and South Asia. *Talinum paniculatum* and *T. fruticosum* are pantropical weeds, often of coastal areas. There are no reports of CAM in *Amphopetalum*, a rare small perennial herb native to seasonal sites in north-west Paraguay.

The monotypic genus Portulacaceae includes ~152 *Portulaca* in six clades. CAM is postulated in the most recent common ancestor of *Portulaca* (Christin *et al*., 2014; Gilman *et al*., 2022), and facultative CAM has been demonstrated in members of all clades (Guralnick *et al*., 2002; Holtum *et al*., 2017*b*; Winter and Holtum, 2017; Winter, 2019; Winter *et al*., 2019*b*). *Portulaca* are C_4_ + CAM plants, because all bar three species exhibit C_4_ photosynthesis (Voznesenskaya *et al*., 2017). The three exceptions are C_3_–C_4_ intermediates, of which *Portulaca cryptopetala* has been shown to exhibit CAM (Winter *et al*., 2019*b*). In contrast to CAM, C_4_ is hypothesized to have evolved separately in at least three clades (Christin *et al*., 2014; Gilman *et al*., 2022). C_4_ photosynthesis is probably absent from stems because they lack Kranz anatomy. In *Portulaca oleracea*, *P. grandiflora* and *P. cryptopetala* (Koch and Kennedy, 1980; Guralnick *et al*., 2002; Winter *et al*., 2019*b*), stems can express low-level CAM, indicating that such plants might simultaneously exhibit C_4_, C_3_ and CAM photosynthesis, C_4_ + CAM in leaves and C_3_ + CAM in stems.

The stem node of *Portulaca* is ~30 Mya (Ocampo and Columbus, 2012; Christin *et al*., 2014), with the most recent common ancestor probably diverging in the early Miocene ~23 Mya (7–43 Mya; Ocampo and Columbus, 2012). An opposite-leaved (OL) *Portulaca* lineage, which arose ~19 Mya (6–35 Mya), possibly in the Old World or Australia, contains ~30 species split between African–Asian and Australian clades plus *Portulaca quadrifida*, a pantropical weed derived from a dispersal event from Africa or Asia (Ocampo and Columbus, 2012).

A geographically separated alternate-leaved (AL) lineage with >100 species arose ~18 Mya (5–32 Mya) in South America and dispersed multiple times to other continents (Ocampo and Columbus, 2012). The AL lineage contains the *oleracea*, *pilosa*, *umbraticola* and the *cryptopetala* clades that, between them, have colonized North South and Central America, Africa, Asia, Australia, the Galápagos and Hawaii. Multiple long-distance dispersals have been postulated for *Portulaca*, but there is no clear dispersal mechanism.

The expression of C_4_ and facultative CAM in *Portulaca* presumably provides a capacity for rapid growth when water is available and a reduction in carbon and water loss when the supply of water is constrained. The induction or reduction in CAM expression following cycles of water supply and water stress points to tight links with environmental triggers, independent of ontogeny, with a rapid switching between C_4_, CAM and back to C_4_ enabling a prompt response to rainfall events. Indeed, a weak constitutive CAM cycle appears transcriptionally and post-transcriptionally upregulated during drought in *Portulaca* (Gilman *et al*., 2022). At least in leaves of *P. oleracea*, CAM and C_4_ carbon fixation occur in the same cells, albeit mutually exclusive genes are involved, and carbon from nocturnally accumulated malic acid might be incorporated into the C_4_ cycle during the light, suggesting substantial integration of the two pathways (Lara *et al*., 2004; Ferrari *et al*., 2020; Gilman *et al*., 2022; Moreno-Villena *et al*., 2022).

Such an ability is of relevance to species that are small, fast-growing with short seasonal life cycles, weedy ecological opportunists of disturbed, pioneer or periodically dry sites, where water supply is ephemeral. Presumably, the seed- or tuber-forming life of the plants is extended, as has been demonstrated experimentally for *Mesembryanthemum crystallinum* (Winter and Ziegler, 1992) and postulated for annual facultative-CAM herbs, such as *Calandrinia polyandra* (Winter and Holtum, 2011).

The perennial Anacampserotaceae, sister to the Portulacaceae (Nyffeler & Eggli, 2010; Wang *et al*., 2019; Moore *et al*., 2018) contains three genera: *Anacampseros* (~59 species), mainly distributed in southern Africa and with disjunct species in the Horn, one species native to Argentina and a diminutive species in arid and seasonally dry Australia (Holtum *et al*., 2016), and two New World monotypic genera, *Grahamia bracteata* and *Talinopsis frutescens* (Nyffeler and Eggli, 2010; Ocampo and Columbus, 2010; Hernández-Ledesma *et al.*, 2015). Old World Anacampserotaceae are thick rooted, sometimes with a caudex, with leaves that tend to be succulent, often on short-lived aerial shoots, or the leaves may be tiny, on a fleshy stem. The New World genera are small, succulent-leaved desert shrubs (Nyffeler *et al*., 2008).

The Anacampserotaceae probably arose in the New World, with a most recent common ancestor age of ~11 Mya (Nyffeler and Eggli, 2010; Ocampo and Columbus, 2010). An early postulated vicariance event separated the North American endemic *Talinopsis frutescens* and the South American *Anacampseros vulcanensis*, *A. coahuilensis*, *A. kutzii* and *Grahamia bracteata*. The Old World *Anacampseros* and the Australian endemic *Anacampseros australiana* are inferred to result from long-distance dispersals from South America.

The δ^13^C values and changes in nocturnal tissue acidities indicate that strong CAM is present in *Anacampseros* (Rundel *et al*., 1999; Guralnick *et al*., 2008; Messerschmid *et al*., 2021), but facultative/inducible CAM has been reported in all three genera of the family (Guralnick and Jackson, 2001; Guralnick *et al*., 2008; Winter and Holtum, 2017). CAM has not been demonstrated in the New World *Anacampseros vulcanensis*, for which δ^13^C values of −23.7 and −24.53 ‰ are available (Guralnick *et al*., 2008; Ocampo and Columbus, 2010).

The Cactaceae is a New World family of ~1800 perennial, mainly succulent species in 144 genera. Distributed from Patagonia to Canada, cacti are conspicuous in semi-arid and arid landscapes, with centres of diversity in Mexico and south-west USA, the central Andes of Peru and Bolivia, and the xeric shrublands and montane-subtropical grasslands of eastern Brazil. The Andean regions of Chile, Argentina and Bolivia are the probable areas of origin of the Cactaceae, which split from their sister Portulacineae ~32 Mya (Edwards *et al*., 2005; Arakaki *et al*., 2011; Hernández-Hernández *et al*., 2014). The extant lineages diverged soon after, ~27 Mya. Cacti from several lineages have invaded biomes in Africa, Asia, Australia and Europe (cf. Mann, 1970).

CAM is present throughout the Cactaceae. In the basal leafy genera, *Leuenbergeria*, *Pereskia* and *Rhodocactus*, leaves and often stems of some species exhibit low levels of nocturnal acidification and CAM cycling (i.e. *Pereskia aculeata* and *P. horrida*, *Leuenbergeria aureiflora*, *L. quisqueyana* and *L. ziniiflora*, and *Rhodocactus sacharosa* and *R. grandifolius*; Rayder and Ting, 1981; Martin and Wallace, 2000; Edwards and Donoghue, 2006; Mauseth, 2006), but others do not (*Leuenbergeria bleo*, *L. lychnidiflora* and *Rhodocactus bahiensis*; Nobel and Hartsock, 1986; Martin and Wallace, 2000). Facultative CAM is known in *Leuenbergeria guamacho* (Edwards and Diaz, 2006). In cacti with photosynthetic stems, strong CAM is invariably present (Nobel and Hartsock, 1986).

The early divergent lineages of Cactaceae do not possess the succulent, essentially leafless photosynthetic stems that are characteristic and morphologically diverse in the later clades (Hernández-Hernández *et al*., 2011). Instead, they are shrubs, trees or climbing vines with persistent leaves and often have stems with bark and dense, fibrous wood. Evolution of the cacti involved transitions from leaf- to stem-based photosynthesis, with the evolution of stem stomata and delayed bark formation anteceding the development of the stem cortex into a photosynthesizing system (Edwards *et al*., 2005). Nevertheless, traits such as a thick stem cuticle, aureoles with spines, prominent stem mucilage cells, hypodermal calcium oxalate druses, high tissue water potentials, shallow roots, rapid response to rainfall events, and highly responsive stomatal behaviour might have facilitated the evolution of the water-storing cactus succulent strategy (Edwards and Donoghue, 2006; Ogburn and Edwards, 2009).

The stem-succulent subfamilies Opuntioideae (stem age, ~19 Mya; crown age, ~9 Mya) and Cactoideae (stem age, ~17 Mya; crown age, ~15 Mya) emerged in the East and Southeast Andes (Hernández-Hernández *et al*., 2014). The Opuntioideae, currently distributed from Canada to southern Argentina, are flat-, spherical- or cylindrical-stemmed, ribbed species of various habits that include geophytes, hemispherical cushions, shrubs, trees and columns. The Cactoideae split into the Cacteae (stem age, ~15 Mya; crown age, ~12 Mya), North American and Mexican globose and barrel-shaped species, with origins in the Chihuahuan Desert, and the core Cactoideae (stem age, ~15 Mya; crown age, ~13 Mya), distributed throughout the New World. The core Cactoideae clades contain ribbed, shrubby, epiphytic, globose, arborescent, epiphytic or columnar forms.

CAM contributed to the radiation of the Cactaceae. Although the ancestral genera with little CAM constitute perhaps only 17 species and the later clades all exhibit strong CAM, it is difficult to disentangle the contribution of CAM to ecological success from other attributes, because different diversification rate estimates for clades originating at similar times suggest different underlying drivers of diversification or, perhaps, differing contributions of the same drivers (Hernández-Hernández *et al*., 2014). The Cactaceae might have originated soon after the Oligocene fall in atmospheric [CO_2_], and their radiation might have coincided with the expansion of aridity in North America during the late Miocene, both climatic features that might be expected to favour selection for CAM (Arakaki *et al*., 2011). A dependence between diversification rate, pollination and growth-form evolution has also been detected (Hernández-Hernández *et al*., 2014).

### CAM in hydrophytes

The ~51 known species of CAM aquatics or hydrophytes include submerged, floating, emergent and semi-terrestrial species (Keeley, 1998*a*). Around 39 species are *Isoëtes* (Isoëtaceae), a lycopsid genus of ~193 species (POWO, 2022). It is assumed that >170 aquatic *Isoëtes* express CAM, because all aquatic *Isoëtes* tested to date exhibit it, but some terrestrial species do not (Keeley, 1983, 1998*a*).

*Isoëtes* is probably the oldest aquatic CAM lineage (Keeley, 1998*a*). *Isoëtes*-like fossils date to the Jurassic and Triassic (Ash and Pigg, 1991) when CO_2_ concentrations might not have limited photosynthesis in terrestrial habitats but CAM-favouring diel changes in dissolved CO_2_ might have occurred in shallow seasonal pools, particularly as temperatures rose (Benton, 2018). Recent evidence suggests that Jurassic/Triassic fossils are stem relatives of extant *Isoëtes* lineages (Wood *et al*., 2020). The latter probably diversified during the last 45–60 Myr, radiating across the globe (Kim and Choi, 2016; Pereira *et al*., 2017; Wood *et al*., 2020). Many relationships uncovered by the recent studies of *Isoëtes* (Wood *et al*., 2020; Larsén *et al*., 2022) contradict intuitive assumptions based on geographical proximity of species such that earlier discussion of the evolution of species and putative amphibious-to-terrestrial transitions and amphibious-to-lacustrine-to-terrestrial transitions (Taylor and Hickey, 1992; Keeley, 1998*a*) need to be reconsidered. In general, hybridization, polyploidy and vegetative growth are common in *Isoëtes*, and dispersal rates and mechanisms are not well understood (Troìa, 2016). A few *Isoëtes*, particularly in Northern Hemisphere temperate areas, have widespread multi-continent distributions (*Isoëtes histrix*, *I. lucustris* and *I. echinospora*), but cryptic species are suspected.

Non-*Isoëtes* CAM aquatics include seven monocots in the families Alismataceae (two *Sagittaria* species), Cyperaceae (one *Scirpus* species) and Hydrocharitaceae (two *Vallisneria* species) and seven eudicots in the families Apiaceae (one *Lilaeopsis* species), Crassulaceae (five species of *Crassula*) and Plantaginaceae (one *Littorella* species) (Keeley, 1998*a*). CAM in two *Ottelia* species (Hydrocharitaceae) remains to be confirmed because the nocturnal increases in titratable acidities reported were measured to pH 8.3, a pH too high to distinguish between malic and other acids (Zhang *et al*., 2014).

Nocturnal acid accumulation in CAM aquatics can be substantial but does not always contribute significantly to autotrophism (Keeley, 1998*a*). In the monocots *Eleocharis acicularis* (Cyperaceae) and *Orcuttia* (Poaceae), low levels of H^+^ accumulate at night, but much of the carbon initially in malate is transferred in the dark to citrate and/or insoluble compounds (Keeley, 1998*a*, *b*). The citrate is unlikely to provide nocturnal storage of fixed CO_2_ because its synthesis from malate is associated with loss of CO_2_ (Lüttge, 1988).

Neither succulence nor plant form distinguishes CAM from non-CAM aquatic plants. Both CAM and non-CAM species have mesophyll succulence ratios > 1 (Keeley, 1998*a*). The internal vacuolar volumes of CAM hydrophytes appear sufficient to dilute malic acid concentrations to levels that are physiologically manageable. The isoetid habit of a rosette of stiff terete leaves (or petioles) containing lacunae attached to a corm, stolon or rhizome that is exhibited by most rooted CAM aquatics (e.g. *Isoëtes*, *Littorella* and *Sagittaria*) is also common in non-CAM aquatics (e.g. *Lobelia dortmanna*, *Sabularia aquatica* and *Eriocaulon septangulare*). This isoetid structure is probably a convergent form in hydrophytes because it confers the following advantages in resource-limited aquatic habitats: (1) small stature; (2) high root-to-shoot biomass ratio; (3) long-lived evergreen leaves; and (4) slow growth (Boston, 1986). A few CAM aquatics have a non-isoetid habit, including *Vallisneria* and *Lilaeopsis*, which have ribbon-like leaves, and *Crassula* species, which, like their CAM terrestrial analogues, are diminutive, caulescent, with short, semi-cylindrical leaves, and often prostrate stems that constitute much of the photosynthetic surface area.

Two functionally important features common to water bodies occupied by CAM hydrophytes are that: (1) carbon supply is limited, either permanently or on a diel (24 h) basis; and (2) they are oligotrophic. CAM aquatics are, in general, poorly represented in mesotrophic lakes and are seldom found in eutrophic waters (Keeley, 1998*a*).

Most CAM aquatics are still-water species of shallow, rain-fed, low-nutrient seasonal pools or deeper lake-like (lacustrine) waters, although species are known from slow-moving shallow streams, ditches, irrigation channels, palustrine habitats and eulittoral zones of freshwater tidal rivers and marshes (e.g. *Crassula* spp., *Isoëtes riparia* and *Sagittaria subulata*; Keeley, 1998*a*).

Seasonal pools inhabited by CAM aquatics in mediterranean climates of California, Western Australia, Chile, South Africa and Spain typically form during winter and spring and are predominantly rain filled. The pools are generally shallow, hence well irradiated, and generally short lived. The substantial dry phase retards establishment of many competitive wetland taxa, but they may support a high biomass of seasonal-pool specialists and cosmopolitan aquatic taxa. The coupling of high plant biomass, high irradiation and poor buffering owing to low nutrient concentrations results in substantial diel (24 h) changes in dissolved [CO_2_], [O_2_] and pH. During daylight, the plant biomass depletes the dissolved [CO_2_] in the water column and particularly across the leaf boundary layer. At night, release of respiratory carbon drives up the ambient [CO_2_]. Ultimately, seasonal pools dry out, and plant leaves die or become aerial.

Lacustrine waters inhabited by CAM plants are generally oligotrophic, with low dissolved mineral contents and inorganic carbon levels one to two orders of magnitude lower than in pools or lakes dominated by non-CAM plants. Such permanently infertile waters are more prevalent at high latitudes or, if in lower latitudes, at high elevations. In these infertile waters, diel fluctuations in CO_2_ availability are small because the vegetation biomass tends to be low (Sand-Jensen, 1989; Sandquist and Keeley, 1990). In seasonal pools, CAM aquatics tend to be only a component of a significant biomass of aquatics present, but in oligotrophic lakes it is not uncommon for the vegetative biomass to be small but dominated by CAM plants, particularly in more acidic waters (Keeley, 1996, 1998*a*).

Amphibious CAM plants are initially submerged but survive the drying out of seasonal pools to continue life as emergents. In *Isoëtes howellii*, *Crassula natans* and *C. aquatica*, submerged parts of leaves retain CAM, but emergent parts and new leaves rely on the C_3_ pathway (Keeley and Busch, 1984; Keeley, 1996, 1998*a*, *b*). The switch from CAM to C_3_ can be associated with enhanced biomass accumulation, presumably because diffusional resistances no longer limit the supply of CO_2_ (Keeley, 1998*a*). Temperate lowland *Isoëtes macrospora* and *Littorella uniflora* not only switch off CAM but also develop functional stomata (Keeley *et al*., 1985; Aulio, 1986; Keeley, 1998*a*), whereas the tropical alpine species *Isoëtes palmeri* and *I. karstenii* (Keeley, 1998*a*) retain CAM and fail to produce stomata.

Strictly terrestrial *Isoëtes* can be non-CAM or C_3_. Species tested from North America, South Africa and Europe appear exclusively C_3_, even when submerged experimentally, and possess leaves with stomata (Keeley, 1983; Richardson *et al*., 1984; Keeley, 1998*a*). In contrast, *Isoëtes andicola*, *I. andina* and *I. novo-granadensis*, three high-elevation tropical species from South America, exhibit strong CAM and have thick cuticles without stomata; this group obtains CO_2_ from sediments (Keeley *et al*., 1984, 1994).

## SUMMARY

The CAM diaspora is global, effectively stretching from pole to pole and from below sea level to 4800 m a.s.l. CAM is present in seedless and seeded vascular plants that have colonized most terrestrial, epiphytic, lithophytic, palustrine and aquatic systems, adopting most plant structural forms and life-cycle strategies. In global terms, CAM perennials with massive stems and/or extremely succulent leaves tend to be strong CAM and restricted to continents where they evolved. They predominately inhabit arid and semi-arid environments in the New World, Southern and Western Africa and Madagascar, with isolated populations also on island outposts, such as Macaronesia and Socotra. Their habitats, mainly in the horse latitudes, have predictable periodic water supplies that are seasonal or, if more frequent, associated with local moist events, e.g. fogs in coastal Namibia and the Atacama. Annuals with CAM are terrestrial, small herbs that tend to exhibit C_3_ + CAM, often with a capacity for facultative CAM. Their short life cycles and ability to fill seed during drier periods enables them to inhabit semi-arid saline and lowland coastal habitats in addition to sheltered micro-sites at higher elevations. Small CAM perennials inhabit moist forests (e.g. *Peperomia*), seasonally arid regions (e.g. many Aizoaceae) and the temperate low and higher elevations of Europe, North America and Russia–Asia (e.g. *Sedum* and *Sempervivum*). Although often very succulent, these small perennials tend not to exhibit strong CAM but are more often C_3_ + CAM. Epiphytes with CAM are found in moist and seasonally dry forests of the New World and Madagascar and in forests of the Palaeotropics, particularly of the Indo-Australasian Archipelago (e.g. orchids, bromeliads and hoyas). Epiphytic groups tend to be species rich, presumably reflecting abundant niche space in the canopy and in landscapes that have altered extensively during their evolution. CAM plants with global distributions tend to be small and weedy, with other adaptations that are enhanced by CAM-assisted drought tolerance, e.g. C_4_–CAM *Portulaca* and *Trianthema* are extremely fast growing and drought tolerant, *Sesuvium* and *Mesembryanthemum* are NaCl tolerant, and the aquatic C_3_ + CAM *Crassula helmsii* grows rapidly and reproduces rapidly via vegetative growth.

For extant CAM lineages, radiation during the mid-Miocene onwards, as the planet dried and [CO_2_] decreased, appears commonplace. Expansion benefitted from the creation of niche spaces as landscapes changed with the emergence of the Andes, closure of the Isthmus of Panama, emerging and submerging landforms in Sundaland, and changing rainfall regimes and desertification in the New World, southern Africa and Australia.

For many clades, particularly tropical ones, estimates of the origins of CAM are clouded by uncertainties associated with the appearance and extinction of taxa during the appreciable periods between stem divergence and the radiation of extant clades (Ramírez-Barahona *et al*., 2020), by uncertain dating, by inadequate knowledge of the presence or absence of low-level CAM, particularly in basal groups, and by proposed mechanistically unspecified or speculative long-distance dispersal events. Insufficient detail is thus available to resolve evolutionary questions, such as: in what order did the biochemical and anatomical components of CAM assemble in different lineages, is there consistent evidence for progressions from C_3_ + CAM to strong CAM states, and does CAM tend to be an early- or late-appearing drought-adaptation trait?

At present, there is little evidence for or against the CAM-biochemistry-first hypothesis (Edwards, 2019) or, indeed, whether the order of the evolution of CAM traits is similar in all lineages. Likewise, evidence is equivocal for either low-level C_3_ + CAM or strong CAM appearing earlier, later or contemporaneously with other drought-adaptation traits. The contention of Males (2016) that ‘…CAM may have repeatedly evolved as a flexible culminating trait in a cascade of adaptations to xeric conditions, rather than precipitating extensive change once it has originated’ is thus still an open and relevant question.

There appear to be broad associations between CAM expression and plant life strategy. Irrespective of plant size, any form of CAM can be present among perennial taxa (including epiphytes), depending upon the lineage and the habitat, although facultative CAM appears less common in epiphytes (but see Fig. 1). In general, strong CAM is less common in plants with shorter life cycles, including annuals. In such plants, CAM tends to be C_3_ + CAM or C_4_ + CAM when constitutive, inducible or facultative. Likewise, in leaves of geophytes that lose their succulent leaves annually, facultative CAM and constitutive C_3_ + CAM are common, but strong CAM is not (cf. Ruschioideae; Winter, 2019).

As a whole, surveys for CAM are patchy across phylogenies and tend to be biased, in that lineages that are not overtly succulent are undersampled, as are cool-climate floras. Testing for CAM in stems of leafy taxa is rare. For most groups mentioned herein, phylogenetic analyses would benefit from the inclusion of more species, more intensive sampling for C_3_ + CAM or C_4_ + CAM, better estimations of hybridization, and analysis of the biochemical category of CAM pathway present. Common bottlenecks to such studies include the time required to survey plants for low levels of CAM and the lack of appropriate molecular markers. Isotopic surveys are quick, but insufficiently informative for the evolutionary questions now being posed. Surveys that measure gas exchange and titratable acidity are accurate but slow and have small throughputs of samples. If suitable molecular markers remain elusive, effort should be accorded to developing methods for the rapid direct measurement of vacuolar or tissue pH. Potential methods include confocal microscopy in conjunction with fluorescent dyes and near-infrared spectroscopy.

Assessment of δ^13^C composition permits the sampling of herbarium specimens for δ^13^C composition and evaluations of CAM plant isotopic values against environmental data in order to create plant–environment response predictions. A major uncertainty to this approach is that, because of the plasticity of CAM expression in many species with CAM, any isotopic value can be the result of different contributions of CAM or C_3_ to carbon gain during the life of the tissue tested. Such uncertainty can be resolved only by exploring whole-plant or leaf gas-exchange responses under a range of temperatures and/or water stress conditions. Such information is available for a few species of *Agave*, cacti, *Clusia*, *M. crystallinum*, *Myrmecodia beccarii* and some Crassulaceae but is not yet available for most categories of CAM plants, including many small perennials and those species that live in conditions at the northern and southern boundaries and the elevational limits of the CAM diaspora.
